# Surfactant-Enabled Nanocarriers in Breast Cancer Therapy: Targeted Delivery and Multidrug Resistance Reversal

**DOI:** 10.3390/pharmaceutics17060779

**Published:** 2025-06-13

**Authors:** Ashirwad Jadhav, Karuppiah Nagaraj

**Affiliations:** 1 Department of Sports Medicine, Saveetha Medical College and Hospital, Saveetha Institute of Medical and Technical Sciences (SIMATS), Saveetha Nagar, Thandalam, Kanchipuram—Chennai Rd, Chennai 602105, Tamil Nadu, India; ashu.6456@gmail.com; 2 Biomedical & Nano-Drug Formulation Laboratory, Department of General Medicine, Saveetha Medical College and Hospital, Saveetha Institute of Medical and Technical Sciences (SIMATS), Saveetha Nagar, Thandalam, Kanchipuram—Chennai Rd, Chennai 602105, Tamil Nadu, India

**Keywords:** surfactant-based drug delivery, breast cancer, polymeric micelles, nanoemulsions, multidrug resistance (MDR), targeted therapy, self-emulsifying drug delivery systems (SEDDSs), clinical nanomedicine, stimuli-responsive nanocarriers

## Abstract

Breast cancer remains a leading cause of cancer-related morbidity and mortality among women worldwide. Its treatment is complicated by molecular heterogeneity and the frequent development of multidrug resistance (MDR). Conventional drug delivery approaches are often limited by poor aqueous solubility, rapid systemic clearance, non-specific biodistribution, and off-target toxicity. This review will critically explore the possibility of surfactant-based drug delivery systems (DDSs) in addressing the constraints of standard breast cancer treatments. It focuses on the mechanisms by which surfactants promote solubility, facilitate cellular uptake, and overcome drug resistance, while also analyzing current therapeutic success and future directions. A thorough review of preclinical and clinical investigations was undertaken, focusing on important surfactant-based DDSs such as polymeric micelles, nanoemulsions, liposomes, and self-emulsifying systems (SEDDSs). Mechanistic insights into surfactant functions, such as membrane permeabilization and efflux pump inhibition, were studied alongside delivery systems incorporating ligands and co-loaded medicines. Pluronic^®^ micelles, TPGS-based systems, biosurfactant-stabilized nanoparticles, and lipid-based carrier surfactant platforms improve medication solubility, stability, and delivery. Genexol^®^ are examples of formulations demonstrating effective use and FDA translational potential. These systems now incorporate stimuli-responsive release mechanisms—such as pH, temperature, redox, immuno- and photodynamic treatment—artificial intelligence treatment design, and tailored treatment advancement, and responsive tailoring. Surfactant-enabled DDSs can improve breast cancer care. Innovative approaches for personalized oncology treatment are countered by the enduring challenges of toxicity, regulatory hurdles, and diminished scalability.

## 1. Introduction

Surfactants are surface-active agents which can reduce tension between the interfaces of two phases, for instance water and oil, because they are amphiphilic compounds containing hydrophilic (water-loving) and hydrophobic (lipid-loving) moieties [1,2]. They can be further categorized into four broad types based on the nature of the polar head group: zwitterionic, anionic, cationic, and nonionic surfactants [3,4,5]. In pharmaceutical sciences, surfactants are of great importance as they improve the solubility, stability, and bioavailability of poorly soluble drugs in water [6]. Due to this self-assembling ability, surfactants can form diverse nanostructures such as vesicles [7,8,9], liposomes, emulsions, and micelles, which are excellent materials for drug delivery [10,11,12]. Certain surfactants exhibit antibacterial [13], anti-inflammatory [14], and anticancer activities, which makes them useful not only as solubilizing agents but as versatile drug delivery systems [15,16,17,18].

Surfactants have attracted increasing attention in oncology because they can penetrate through a series of barriers in cancer treatment, particularly breast cancer [19,20]. They enhance cellular uptake, facilitate the ease of drug passage across biological membranes, and can alter membrane fluidity to enhance drug penetration into tumor tissues [21,22,23,24]. In addition, certain surfactants, such as D-α-tocopheryl polyethylene glycol succinate (TPGS), have been demonstrated to inhibit efflux pumps such as P-glycoprotein (P-gp), a significant contributor to the multidrug resistance (MDR) of cancer cells [25,26]. Surfactants stand as vital stakeholders in the design of the next generation of drug delivery systems through their role as both a therapeutic modulator and delivery booster [27,28]. In addition to improved pharmacokinetics and biodistribution, their presence in nanocarriers for breast cancer therapy also brings with it the hope of targeted and controlled release, which would minimize systemic toxicity and maximize therapeutic efficiency [29,30,31].

Breast cancer continues to be one of the most common and far-reaching forms of cancer worldwide, with millions of women being diagnosed annually [32]. Breast cancer is a heterogeneous disease with varied clinical, molecular, and genetic differences that make treatment difficult. Invasive ductal carcinoma (IDC), invasive lobular carcinoma (ILC), and less common types such as inflammatory breast cancer and Paget’s disease are the primary forms of breast cancer [33]. These tumors are divided based on where they are and what the cells look like, which impacts treatment and prognoses. Breast cancer is a heterogeneous disease with numerous subtypes at the molecular level, including triple-negative breast cancer (TNBC), hormone receptor-positive (HR^+^) breast cancer, and human epidermal growth factor receptor 2-positive (HER2^+^) breast cancer, each with its own implications for treatment. Notwithstanding advances in targeted treatment, early detection, and diagnostic techniques, breast cancer remains a leading cause of cancer-related morbidity and mortality.

The standard multimodal treatment modalities for breast cancer include surgery, radiation, chemotherapy, hormone therapy, targeted medicines, and, in some situations, immunotherapy [34,35]. Therapy outcomes, however, remain unpredictable, especially in individuals with aggressive subtypes like TNBC, which lack obvious molecular targets. Chemotherapy, the cornerstone of treatment for many kinds of breast cancer, frequently faces problems such as systemic toxicity, drug resistance, and limited drug penetration into the tumor [36]. Radiation- and chemotherapy-induced side effects also affect the quality of life for many patients 37, emphasizing the importance of using novel approaches to increase the accumulation of therapeutic drugs in the cancer region. The therapeutic usefulness of traditional drug delivery systems against breast cancer is often reduced by several issues [37]. These include the poor water solubility of hydrophobic drugs, fast clearance in the bloodstream, the lack of specificity for their distribution, and a failure to focus on their sites of tumor correctly. Due to the systemic delivery of most of the chemotherapeutic agents, high doses in non-targeting tissues can cause severe toxic effects [38,39,40]. In addition, the hydrophobicity of most anticancer drugs complicates their formulation for effective distribution. In addition, both the tumor microenvironment (TME) and the blood–brain barrier (BBB) can further limit drug penetration, creating a challenge to achieve therapeutic concentrations of the drug in the area of the tumor [41].

Surfactant-based drug delivery systems (DDSs) are a viable alternative to conventional drug administration methods that address many of the constraints associated with standard treatments [42,43,44]. Surfactants, which are amphiphilic compounds with both hydrophilic and hydrophobic sections, can greatly increase the solubility and bioavailability of medicines that are poorly water soluble [42]. These systems can create a variety of nanostructures, including nanoparticles, emulsions, liposomes, and micelles, which encapsulate therapeutic compounds, preserve them from degradation, and enhance their capacity to pass biological barriers [45,46,47]. Furthermore, surfactant-based DDSs can be designed for regulated drug release, enhancing therapeutic outcomes while minimizing side effects [48,49]. Improving the pharmacokinetics and biodistribution of drugs is one of the primary advantages of surfactant-based DDSs, and they are capable of ensuring that greater amounts of drug are delivered to the tumor site by restricting premature drug release and reducing the agglomeration of hydrophobic drugs [50]. In addition, surfactants can be functionalized to bind with specific receptors on cancer cells, increasing drug delivery strength and specificity. For example, ligand- or antibody-responsive overexpressed receptors on tumor cells may be assimilated into surfactant-based carriers for enhancing targeted delivery and reducing off-target injury [51,52]. Furthermore, surfactant-based DDSs provide the opportunity to combine multiple therapeutic agents onto one platform, i.e., immunotherapy, gene therapy, and chemotherapy [53]. This combination approach can improve treatment efficacy and address the complex and diverse character of breast cancer by addressing several facets of tumor biology at once. For instance, simultaneous administration of a chemotherapeutic drug with an immune checkpoint inhibitor or small interfering RNA (siRNA) in a surfactant-based DDS may not just reduce tumor cell proliferation but also modulate the immune system to boost anti-tumor activity [54].

Surfactant-based DDSs can potentially enhance patient compliance as well as facilitate better drug delivery. Standard chemotherapy protocols often require many cycles of treatment, which can be stressful and result in prolonged hospitalization stays [53]. On the other hand, surfactant-based systems can offer a drug’s prolonged release over time, lowering dosage frequency and enhancing patient adherence to the prescribed course of treatment. This is particularly crucial in cases of breast cancer, where the patient’s quality of life is crucial and therapy may be drawn out. Additionally, because of their adaptability, surfactant-based DDSs can be used in a wide variety of medicinal formulations, from small-molecule medications to biologics such peptides, nucleic acids, and monoclonal antibodies [55]. Due to their flexibility, surfactant-based systems can be applied in various treatment regimens, from systemic administration to targeted therapy, making them more promising for the treatment of breast cancer. Therefore, surfactant-based drug delivery methods offer a suitable alternative to conventional drug delivery methods in the treatment of breast cancer. These innovative technologies can significantly enhance the therapeutic value of anticancer drugs by addressing key issues like systemic toxicity, poor tumor targeting, and low drug solubility. A major leap towards personalized medicine can be taken with the development of surfactant-based DDSs that are tailored to specific patient needs and breast cancer types, giving a safer and more effective way of controlling the disease. The development, mechanism, and therapeutic potential of surfactant-based drug delivery methods in the therapy of breast cancer will be discussed within this review, highlighting their potential to overcome current therapy challenges and unlock more effective treatments.

**Figure 1:**
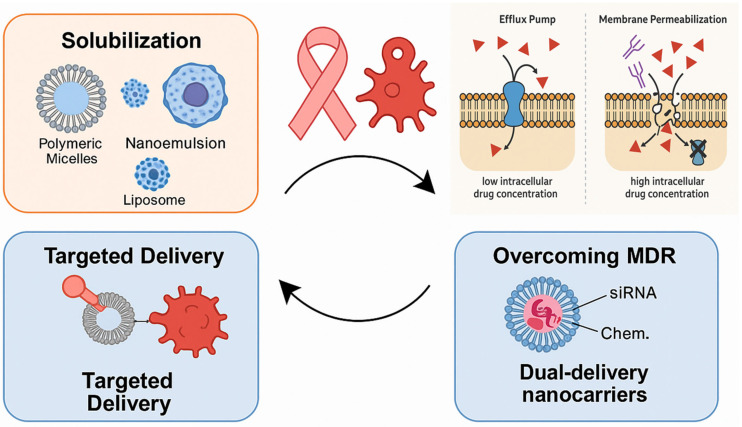
Overview of surfactant-based drug delivery in breast cancer. Pink ribbon (center-top)—typically signifies breast cancer awareness. Red spiky blob (center-top and bottom-left)—suggests a cancer cell or tumor.

The primary objective of this review is to explore the role of surfactant-based drug delivery systems in breast cancer treatment, highlighting their types, therapeutic value, molecular mechanisms, and challenges they aim to overcome. The research aims to explore how surfactants enhance drug permeability and facilitate the reversal of drug multidrug resistance (MDR) through processes like solubilization, micellization, and membrane fluidization, which yield improved outcomes in treating breast cancer (Figure 1). In addition, this review aims to explore a number of surfactant-based systems, such as polymeric micelles, nanoemulsions, and liposomes, and determine the efficacy of each in delivering gene therapy, biologics, and chemotherapeutics. The FDA-approved or clinical-stage products that make use of surfactants, targeted delivery, and the biocompatibility of the systems will also be discussed in the review. In addition to investigating the current challenges and future advancements of surfactant-based systems in precision medicine, regulatory matters, and AI-based design, the research will also depict current innovations and trends.

## 2. Methodology

To ensure scientific rigor and relevance, the present study employed a systematic and thorough literature search strategy. High-profile scientific databases like PubMed, Scopus, Web of Science, and Google Scholar were searched for English-language, peer-reviewed articles from 2010 to 2025. A thorough list of keyword sets together with Boolean operators was employed, i.e., “surfactants”, “drug delivery systems”, “breast cancer”, “multidrug resistance”, “nanocarriers”, “micelles”, “liposomes”, and “TPGS”. The search was limited to preclinical research, clinical trials, FDA-approved drugs, and mechanism-based research on surfactant-based drug delivery systems in the treatment of breast cancer. Original research articles, reviews, and clinical publications with substantial technical advances, mechanistic insights, or translational potential were considered for inclusion. Non-English publications, editorials, commentaries, and review papers that did not contain primary data were excluded. Each study was evaluated rigorously for design robustness, usefulness to clinical application, and reproducibility of results. Selected papers were examined for data on surfactant types, drug delivery augmentation methods, formulation tactics, therapeutic effects, and safety profiles. The retrieved data were categorized into topical areas to give a consistent summary of current trends, difficulties, and future prospects in the field of surfactant-based drug delivery for breast cancer treatment.

## 3. Mechanistic Role of Surfactants in Drug Delivery

Surfactants, in the process of micelle formation, enhance drug delivery and reduction in multidrug resistance. These systems promote controlled release mechanisms and enhance targeted therapy, especially in cancer therapeutics. Nanomaterials also aid in the solubility, stability, and general therapeutic effect of drugs, maximizing their delivery and performance (Table 1). Given their numerous applications in drug delivery systems, surfactant amphiphilic agents have received a lot of attention in the pharmaceutical sciences [56,57,58]. Micellization and solubilization are two of the most fundamental mechanisms by which surfactants enhance drug bioavailability [59,60,61,62,63]. At concentrations above their critical micelle concentration (CMC), surfactants form micelles with hydrophobic cores that trap poorly water-soluble drugs [64,65,66]. Medication solubility is enhanced, enzymatic degradation is avoided, and controlled release is facilitated by this micellar encapsulation [67]. Tween 80, Cremophor EL, and Pluronic F127, for instance, have been widely used to formulate hydrophobic anticancer drugs like docetaxel and paclitaxel, significantly enhancing their systemic bioavailability and aqueous solubility [68,69,70,71,72,73]. Further, such micelles can be used as nanoscale drug carriers for enhancing drug uptake across the gastrointestinal system and lymphatic uptake [72].

Surfactants improve permeability and micellar solubilization by controlling the dynamics and structure of biological membranes [70]. Surfactants that alter the lipid architecture in the stratum corneum or epithelial barriers, such as sodium lauryl sulfate (SLS), bile salts, and Labrasol, result in improved transcellular and paracellular transport [74,75]. These substances improve the permeability of lipid bilayers by fluidizing the membrane through their interaction with membrane phospholipids [76,77,78]. Nonionic surfactants, such as polyoxyethylene ethers, have been shown in the literature to improve the passage of both small and large molecules across epithelial cells while decreasing the order of lipid chains in membranes [79,80,81]. This characteristic is especially advantageous for applications involving transdermal and transmucosal medication administration.

### 3.1. Solubilization and Micellization

The ability of surfactants to form micelles and enhance the water solubility of hydrophobic drugs has been a subject of extensive research [68,69,82,83]. When the concentration of surfactant is greater than the critical micelle concentration (CMC), the micelle experiences micellization, which allows the hydrophilic structures to engage with the aqueous phase while the hydrophobic sections comprise the core of the micelle [72,84,85,86,87]. Based on Zhang et al. (2008), the anticancer drug paclitaxel, which is not very soluble in water, was made significantly more soluble by polymeric micelles of Pluronic block copolymers, which enhanced its therapeutic effectiveness [88,89,90,91]. Yoo and Park, 2001 demonstrated that doxorubicin-loaded micelles formed by PEG-poly(aspartate) block copolymers which enhance tumor accumulation using the permeable and retaining effect (EPR effect), which increased solubility and circulating time in vivo [92]. Similarly, Torchilin et al. (2001) verified that PEG-shelled stealth micelles improved drug bioavailability through improved plasma stability and decreased mononuclear phagocytic system recognition [42].

According to Jhaveri and Torchilin (2014), amphiphilic block copolymer micellar systems may be able to administer a variety of hydrophobic medications, which would increase the efficacy of combination therapy [93]. Additionally, Zhu and Jiang (2007) examined the structure–activity connections of different micelle-forming copolymers and came to the conclusion that drug loading and release kinetics were directly influenced by hydrophobicity, block length, and ionic nature [94]. According to Cai et al. (2011), Micelles that are functionalized with targeting ligands like folate or RGD peptides showed improved cellular uptake and targeted drug delivery to cancer cells [95]. Targeted systems decreased off-target toxicity but improved cytotoxicity in cancer cells. Polyhistidine segments were also used by Zhang et al. (2022) to create pH-sensitive micellar structures that break down in acidic tumor settings, enabling regulated medication release [96]. Surfactant-based micelles offer a number of benefits, including the ability to achieve targeted drug distribution, improve pharmacokinetics, and solubilize hydrophobic anticancer medications [84,85,86,87]. As potentially useful instruments for getting around obstacles in traditional chemotherapy formulations, these technologies are constantly developing.

### 3.2. Permeation Enhancement and Membrane Fluidization

Numerous studies have been conducted on the role of surfactants as permeation promoters in transdermal and mucosal drug delivery systems. Surfactants facilitate drug penetration through biological membranes by changing the fluidity and morphology of lipid bilayers [97,98,99]. Surfactants, such as polyoxyethylene ethers and sodium lauryl sulfate (SLS), alter the stratum corneum’s lipid structure and enhance skin permeability, as reported by Lemery et al. (2015) and other scientists [100,101,102]. Rege et al. (2022) discovered that nonionic surfactants, such as Tween 80, increase intestinal membrane permeability by fluidizing the lipid bilayer and momentarily breaking tight junctions [103]. Shah (2004) discovered that bile salt-type surfactants, such as sodium taurocholate, improved paracellular transport by opening up tight junctions in the nasal epithelium, which in turn greatly increased the bioavailability of peptide medications [104].

Elnaggar et al. (2014) also investigated the use of lecithin and polysorbate 80 in topical formulations [105]. Without sacrificing membrane integrity, they discovered that both surfactants improved drug flux and reduced membrane resistance. Furthermore, Sebaaly et al. (2021) hypothesized a synergistic impact after studying chitosan-coated liposomes with surfactant additives and discovering a notable improvement in mucosal penetration throughout the gastrointestinal system [106]. Mixed micelles of bile salts and phosphatidylcholine improved intestinal absorption of cyclosporin A [107,108], a poorly absorbed immunosuppressant, by increasing its membrane permeability and solubility. According to Cavanagh (2018), surfactants that fluidize the membranes of epithelial cells can improve drug absorption by accelerating the processes of endocytosis and transcytosis [109]. These results all point to the likelihood that surfactants facilitate new transport pathways and improve medication permeability by fluidizing lipid bilayers. However, a rigorous tuning process is required for clinical application in order to strike a compromise between membrane safety and permeability increase [110].

### 3.3. P-glycoprotein (P-gp) Inhibition to Reverse MDR

The efficacy of most chemotherapeutic agents is greatly compromised by multidrug resistance (MDR) in cancer, which is often due to overexpression of P-glycoprotein (P-gp). By inhibiting P-gp’s efflux activity, surfactants have emerged as effective P-gp inhibitors that enhance intracellular drug accumulation [111,112]. Pluronic block copolymers have been reported by Kabanov et al. (2002) to enhance membrane fluidity and inhibit P-gp ATPase activity, which can sensitize drug-resistant tumor cells [113,114,115]. According to Batrakova et al. (2003), Pluronic P85 suppressed drug resistance in MDR tumor cell lines by blocking mitochondrial function and ATP depletion, which in turn hindered drug efflux through P-gp [116,117,118,119]. Similarly, nonionic surfactants such as polysorbate 80 and Cremophor EL enhanced paclitaxel oral bioavailability in rats without inhibiting P-gp activity, according to Collnot et al. (2006) [120,121].

**Figure 2:**
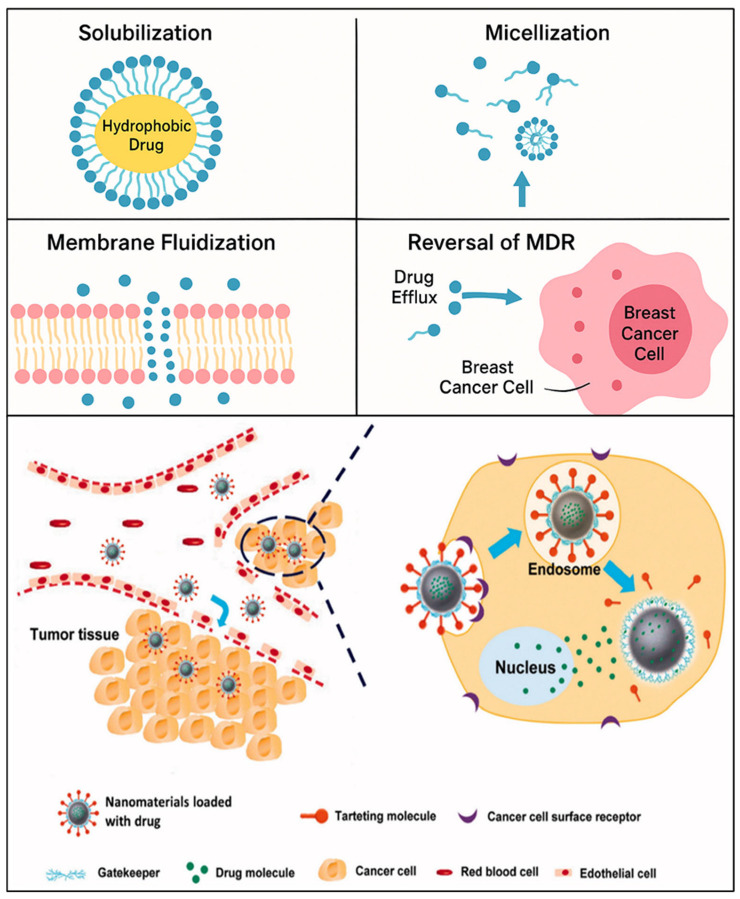
Mechanisms of surfactant-based drug delivery in breast cancer and strategies to overcome MDR.

According to Collu and Cascella (2013), who investigated the relationship between surfactants and membrane-bound efflux pumps, surfactants can change the structure of P-gp, preventing it from binding substrates [122]. Furthermore, doxorubicin and the P-gp inhibitor verapamil were synergistically delivered by Reddy et al. (2004) using surfactants in liposomes, which restored resistance in cancer mice [123,124]. Bile salts, polysorbates, and PEG-derived surfactants demonstrated high P-gp inhibitory activity with little cytotoxicity, according to Kecman et al. (2020), who analyzed a number of surfactant classes [125]. Furthermore, Cremophor EL was shown by Dintaman and Silverman (1999) to enhance vinblastine entrance in Caco-2 cells in a concentration-dependent manner while suppressing P-gp transport activity [126]. Therefore, surfactants modify the membrane environment and inhibit P-gp function directly, both of which are responsible for MDR reversal (Figure 2). One potential way to enhance intracellular drug loading and recover the efficacy of chemotherapeutic drugs in drug-resistant malignancies is their incorporation into nanocarrier systems.

**Table 1:** Nanomaterials for drug delivery and cancer therapy.

Type of Nanoparticle/Nanomaterial/Biosensor	Coated Materials	Description of the Study	Mechanistic Insights	Applications	Ref.
Pluronic (PF127) micelles	Hydrophobic drugs	Drug delivery through thermoresponsive Pluronic micelles	Thermoreversible gelation, micelle encapsulation	Cancer treatment	[68,69]
Pluronic P105/F127 micelles	Docetaxel	Targeting Taxol-resistant lung carcinoma	Mixed micelle optimization for resistance bypass	Cancer chemotherapy	[70]
Pluronic micelles	Anticancer drugs	Solubilization and release of hydrophobic drugs	Temperature- and concentration-dependent release	Anticancer treatment	[71]
Mixed Pluronic micelles	Paclitaxel	Treatment of multidrug-resistant tumors	Enhanced permeability and retention (EPR) effect	Overcoming drug resistance	[73]
Thermoreversible Pluronic^®^ F127 hydrogel (Modular Compact Rheometer (MCR 302), Anton Paar GmbH, Graz, Austria)	Paclitaxel-liposomes	Controlled drug delivery through hydrogel system	Liposomal entrapment in reversible gel	Targeted delivery, cancer	[74]
Nano-sized delivery systems	Various hydrophobic drugs	Parenteral formulation strategies	Nanoscale encapsulation for improved solubility	Oncology and therapeutics	[75]
Silver nanoparticles (AgNPs) via metallo-surfactants	AgNPs assisted by metallo-surfactants	Synthesis of silver NPs by metallo-surfactants for selective detection of amino acids	Colorimetric sensing using plasmon resonance shift in AgNPs; potential interaction with cancer-related metabolites	Potential diagnostic agent for amino acids associated with cancer metabolism	[87]
Metallo-surfactants with π-conjugated ligands	Intercalated with yeast tRNA	Examined hydrophobicity and ligand size on interaction with yeast tRNA (model for cancer RNA studies)	π–π stacking and intercalative binding with RNA; mimicry of drug–RNA interactions	Insights into RNA targeting in cancer chemotherapy	[88]
Polymeric micelles (Pluronic P105)	Paclitaxel-loaded micelles	Explored pharmacokinetics and biodistribution of micelle-encapsulated paclitaxel	Improved solubility, passive targeting through EPR effect	Targeted delivery of cancer drugs (Paclitaxel)	[91]
PLGA–PEG micelles	Doxorubicin-conjugated	Biodegradable micelles to deliver doxorubicin	Cleavable linkers release drug intracellularly; enhanced circulation time	Targeted tumor chemotherapy	[92]
Multifunctional polymeric micelles	siRNA and drug co-loaded	Delivery platform for co-administration of siRNA and chemotherapeutics	Overcomes MDR, RNA silencing + drug action synergy	Gene silencing + chemotherapy in malignancies	[93]
Chitosan-based micelles with RGD peptide	Chitosan, PLGA, RGD ligand	Micelles specifically target integrin-overexpressing tumor cells	Ligand–receptor binding (αvβ3 integrin) increases cellular uptake	Active tumor targeting and delivery of anticancer drugs	[95]
Poly(L-histidine)-based nanovehicles	pH-sensitive polyhistidine	pH-responsive drug delivery smart nanovehicles	Histidine protonation in acidic tumor environment releases drug	Controlled release in tumor tissue	[96]
Dual P-glycoprotein and CA XII inhibitors	Small-molecule inhibitors	Designed dual drugs to reverse P-gp-mediated MDR	Inhibits both P-gp efflux pump and CA XII enzyme activity	MDR reversal in cancer treatment	[110]
Molecular P-gp inhibitors	Tariquidar derivatives	Designed tariquidar analogs to combat MDR	Blocks P-gp and BCRP efflux transporters	Sensitization of chemotherapy in resistant cancers	[112]
Pluronic block copolymers (P85)	Micellar delivery system	Improved intracellular delivery through inhibition of P-gp	Alters membrane fluidity; inhibits efflux pumps	Overcomes MDR in cancer cells	[116]
Pluronic block copolymers	P85, P105	Explored optimal structure for efflux inhibition	Micellar stabilization + efflux pump suppression	Effective delivery of drugs to MDR tumors	[117]

## 4. Types of Surfactant-Based Systems in Breast Cancer Therapy

Surfactant-coated nanoparticles and biosensor systems improve controlled release, drug targeting, and solubility for successful cancer treatment. The systems also overcome issues such as drug resistance and tumor specificity (Table 2). Given that surfactant-based systems can enhance drug solubility, stability, and drug targeting, they are being researched increasingly for the delivery of chemotherapeutic drugs in breast cancer treatment [127,128,129,130]. These platforms, which provide unique advantages in addressing the challenges associated with conventional chemotherapy, typically include polymeric micelles, nanoemulsions, liposomes, self-emulsifying drug delivery systems (SEDDSs), and biosurfactant-based nanocarriers [131,132,133,134]. As noted by Junnuthula et al. (2022), polymeric micelles, such as Pluronic micelles incorporating doxorubicin (DOX) or paclitaxel (PTX), have been shown to enhance drug solubility and provide controlled release, resulting in enhanced therapeutic efficacy and reduced toxicity [135,136,137,138]. Surfactants and oils come together to produce nanoemulsions and microemulsions, which are advantageous in solubilizing hydrophobic drugs [139,140] and retaining their stability in circulation [141].

Based on studies by Hong et al. (2014), PTX-loaded nanoemulsions can enhance the drug accumulation in tumor tissues, thus enhancing the drug’s anticancer effects [142]. Another highly explored system is surfactant-stabilized liposomes, which enhance the bioavailability of hydrophobic drugs such as DOX and possess the ability to encapsulate hydrophobic and hydrophilic drugs such that different agents may be combined for synergistic therapy [143]. By combining oils, co-surfactants, and surfactants, self-emulsifying drug delivery systems (SEDDSs) increase the oral bioavailability of poorly soluble medications by creating microemulsions that react with aqueous conditions. According to research by Jayaswal et al. (2024), PTX-loaded SEDDS greatly improved the solubility and absorption of drugs [144]. Lastly, research by Gonçalves et al. (2018) suggests the use of biosurfactant-based nanocarriers, which offer biodegradable and biocompatible drug delivery formulations and are made of natural surfactants like rhamnolipids [145]. They are less toxic than conventional synthetic surfactants and have been reported to be effective in encapsulating DOX for the treatment of breast cancer. The safety, targeting, and efficacy of therapies for breast cancer might all significantly be improved through these surfactant-based systems.

### 4.1. Polymeric Micelles (e.g., Pluronic Micelles with DOX, PTX)

Polymeric micelles are a well-established family of surfactant-like systems for the delivery of drugs, especially poorly soluble drugs such as paclitaxel (PTX) [146,147] and doxorubicin (DOX). Polymeric micelles consist of amphiphilic block copolymers, which self-assemble to form core–shell structures in water environments. While the shell consists of hydrophilic segments that ensure solubility in biological fluids, the core is typically hydrophobic, providing a stable environment for the encapsulation of hydrophobic drugs [148,149,150]. The role of pluronic block copolymers, including pluronic F127 and P85, in drug delivery is one of the most widely studied. Numerous chemotherapeutic medications, including PTX and DOX, can be efficiently encapsulated by pluronic-based micelles, according to experiments [151,152,153,154,155,156]. For example, Yadav et al. (2023) found that PTX-loaded Pluronic micelles decreased systemic toxicity while increasing PTX’s anticancer activity in murine breast cancer models [157]. Furthermore, by improving the distribution of PTX to tumor tissues, these micelles improved the therapeutic results in comparison to free PTX. According to a study by Majumder et al., 2020, the micellar form of DOX showed decreased cardiotoxicity and increased anticancer efficacy in vivo, demonstrating that these polymeric micelles not only improved solubility but also markedly increased DOX’s bioavailability. Additionally, research has looked into polymeric micelles’ potential to combat multidrug resistance (MDR), a significant barrier to breast cancer treatment [158]. Pluronic P85 micelles can increase the lethal action of DOX in drug-resistant MCF-7/ADR cells by inhibiting the function of P-glycoprotein (P-gp), an efflux pump that reduces intracellular drug levels, as per a study by Colakyan et al. (2006) [159,160]. Pluronic micelles [161] can reverse P-gp-mediated MDR by changing the membrane properties of the efflux pump, as per another study by Rathod et al. (2022). Such micelles are potential carriers in chemotherapeutic regimens for breast cancer due to their dual capacity to entrap drugs and inhibit drug resistance mechanisms.

Among the several benefits of Pluronic micelles is their biocompatibility and low toxicity profile [162]. Gothwal et al. (2016) examined the safety of Pluronic micelles and found that, in in vitro and in vivo models, they exhibited negligible toxicity, even with high doses [163]. In developing chemotherapeutic formulations, this is an important consideration because minimizing the detrimental effects of chemotherapy drugs can improve patient compliance and quality of life. The selectivity of Pluronic micelles for breast cancer cells can be further enhanced by targeting them through functionalization with targeting ligands [164,165]. The functionalization of Pluronic micelles with folic acid is a notable case in point. This further increases the selective delivery of anticancer drugs by targeting overexpressed folate receptors on most breast cancer cells. Some recent developments in the application of polymeric micelles for breast cancer treatment have also centered on the co-delivery of two or more drugs [166,167,168,169,170]. A dual-drug-loaded micelle system consisting of PTX and DOX was developed by Wang et al. (2016), and it has enhanced antitumor activity and synergistic effects in the treatment of breast cancer [171]. Since the two chemotherapeutic medicines were being administered simultaneously, the dosages required for each drug were lowered, decreasing the risk of side effects associated with higher dosages. Furthermore, this approach had an increased tumor growth suppression, highlighting the importance of combination drugs in improving therapeutic outcomes. Polymeric micelles are still being explored as a potential nanocarrier for breast cancer treatment in the future [166,167,168]. The objective of current research is to enhance their stability, drug-loading capacity, and ability to penetrate barriers such as poor tumor penetration and drug resistance. The integration of stimuli-sensitive elements (e.g., temperature- or pH-sensitive) within micellar drug delivery has the capability of enhancing therapeutic outcomes through controlled drug release at the tumor site [93].

Pluronic micelles, or amphiphilic block copolymers, provide a diverse platform for drug administration, particularly in cancer therapy [162,163,164,165]. Pluronic micelles, when functionalized with targeting ligands such as antibodies, peptides, or small molecules, can preferentially target specific receptors on breast cancer cells, such as HER2 or folate receptors [166,167]. This tailored administration increases drug accumulation in tumor tissue, boosting treatment efficacy while reducing systemic exposure. As a result, higher concentrations of therapeutic drugs can be confined to the tumor, which not only boosts the medicine’s efficiency but also lowers the likelihood of unpleasant side effects that are normally caused by nonspecific drug distribution. However, there are many limits to the therapeutic use of ligand-functionalized Pluronic micelles [168]. These include batch-to-batch variability in micelle formation, which can have an impact on consistency and reproducibility, as well as potential immunogenicity caused by the presence of foreign ligands. Furthermore, while targeting ligands enhance specificity, micelles still struggle with poor penetration into solid tumors, which can limit drug delivery to deeper tumor locations [169]. Furthermore, premature drug release from micelles in circulation can shorten the therapeutic window and result in off-target consequences [170,171]. To address these problems, recent efforts are focusing on producing stimuli-responsive micelles that release the drug selectively in the tumor microenvironment, improving penetration and increasing the therapeutic outcome while lowering the danger of premature drug leakage and adverse effects.

### 4.2. Nanoemulsions and Microemulsions

Due to their physicochemical differences, such as high surface area, tiny droplet size, and stability in biofluids, microemulsions and nanoemulsions are surfactant nanocarriers that have been explored in drug delivery [172,173,174,175,176]. Surfactants, oils, and co-surfactants spontaneously assemble to create these systems, which yield clear, stable, and thermodynamically stable formulations [177,178]. Nanoemulsions have been widely investigated for the administration of lipophilic medications, such as paclitaxel (PTX), in the treatment of breast cancer [179]. According to Jampilek and Kralova (2019), PTX-loaded nanoemulsions made of surfactants such as Tween 80 and Labrasol demonstrated improved drug solubilization and markedly increased drug bioavailability [180] in comparison to standard formulations. One of the main advantages of nanoemulsions is their capacity to improve medication penetration into tumor tissues. Jain et al. (2012) discovered that paclitaxel-loaded nanoemulsions improved cellular absorption and therapeutic efficacy in [180] breast cancer cells (MCF-7). The small droplet size and surfactant-mediated membrane fluidization, which are advantageous for drug transport across lipid bilayers, were credited by the researchers with the enhanced cellular internalization. Furthermore, it was discovered that surfactant-stabilized nanoemulsions were more stable, which is essential for preserving the drug’s therapeutic effectiveness while it is in use and directed against tumors. Because of their minuscule droplet sizes, microemulsions have also been studied as drug delivery systems for both hydrophilic and hydrophobic medications.

DOX and PTX microemulsion formulations considerably improved drug solubility and bioavailability during the treatment of breast cancer, as reported by Shaikh et al. (2021) [181]. These systems also demonstrated improved tumor targeting because they could take advantage of the EPR effect, which happens when nanoparticles prefer to aggregate in tumor tissues because of their leaky vasculature [182]. PTX-loaded microemulsions improved tumor shrinkage and extended survival rates in tumor-bearing mice in in vivo experiments when compared to free PTX, suggesting the potential of microemulsions for the treatment of breast cancer. The propensity of nanoemulsions and microemulsions to leak medications and become unstable while stored is one of the main obstacles to their clinical use. Recent studies have focused on using biocompatible surfactants, like PEGylated surfactants, to stabilize these systems. These surfactants improve formulation stability and circulation times. According to a study by Suk et al. (2016), PTX-loaded nanoemulsions stabilized by PEGylated surfactant showed improved pharmacokinetics and decreased toxicity [183]. Additionally, PEG chains added to the surfactants decreased the compositions’ immunogenicity, increasing their potential for use in a clinical setting. Combination therapy becomes a possibility due to the capacity of nanoemulsions and microemulsions to co-encapsulate multiple therapeutic agents. Singh et al. (2017) demonstrated the usage of a nanoemulsion technology for the co-delivery of paclitaxel and DOX to improve therapeutic effect against breast cancer cells [184]. Combination treatment using DOX and PTX within one nanocarrier provided greater killing of tumor cells and minimized treatment resistance risk. In the case of breast cancer treatment, this synergistic drug delivery method has been promising in overcoming the limitations of monotherapy [185]. Aside from their therapeutic applications, nanoemulsions and microemulsions have the advantage of being easily modifiable to a number of delivery routes, including topical, intravenous, and oral routes. They are ideal for enhancing the bioavailability of drugs that are poorly soluble due to their ability to provide stable, nanosized droplets under physiological conditions. To further enhance the targeting ability and therapeutic index of nanoemulsion- and microemulsion-based drug delivery systems in breast cancer treatment, future research should focus on optimizing the surfactant compositions and droplet sizes.

### 4.3. Liposomes Stabilized by Surfactants

Liposomes are nanocarriers with multifunctional properties composed of lipid bilayers that have the ability to encapsulate both hydrophilic and hydrophobic drugs. Due to their ability to increase drug solubility, protect drugs from degradation, and target cancer cells, they are being researched intensively for the treatment of breast cancer [185,186]. Liposomes’ stability and drug-release characteristics frequently need to be improved in order to increase their therapeutic usefulness. To get around these problems, liposome formulations have added surfactants to improve drug loading, prolong circulation periods, and stabilize the lipid bilayer. Liposome formulations, including surfactants like polysorbate 80, showed markedly improved stability and regulated drug release in a study by Forouhari et al. (2022), making them more efficient in delivering chemotherapeutic medicines like DOX to breast cancer cells [187]. Because surfactant-stabilized liposomes reduce lipid vesicle aggregation and produce a more uniform size distribution, they also have increased bioavailability For example, Patel et al. (2022) demonstrated that surfactant-stabilized liposomes loaded with PTX, such as Poloxamer 188, demonstrated improved tumor development and circulation half-lives in animal models of breast cancer [142]. Surfactant-modified liposomes improved therapeutic efficacy and drug deposition at the tumor site by taking advantage of the EPR effect. Additionally, adding surfactants like PEG produced a “stealth” liposome preparation that prevented early clearance and decreased reticuloendothelial system (RES) recognition [188].

Surfactants may assist liposomes in overcoming multidrug resistance (MDR) of breast cancer as well as enhancing stability and bioavailability. P-glycoprotein (P-gp) efflux pumps, which pump drugs out from cancer cells, are often the reason for MDR, a primary reason for chemotherapy failure. Based on studies conducted by Yang and Liu (2016), surfactants such as polysorbate 80 can enhance the intracellular level of chemotherapeutic drugs by suppressing P-gp activity [189]. These findings suggest that surfactant-modified liposomes hold great promise for lowering MDR in the treatment of breast cancer because they may be used both as drug resistance modulators and hydrophobic drug carriers. In addition, controlled and targeted drug delivery can be achieved through surfactant-stabilized liposome engineering. The liposomes may selectively target tumors by functionalizing their surface with targeting ligands such as folic acid, which is attached to folate receptors overexpressed on most breast cancer cells. Helmy et al. (2022) explored the use of folate-conjugated liposomes encapsulating DOX and found that these liposomes greatly enhanced tumor accumulation and cellular uptake in vitro and in vivo [190]. This strategy raises the drug’s therapeutic index while lowering the systemic toxicity associated with conventional chemotherapy.

Another important advantage of surfactant-stabilized formulations is the controlled release of drugs from liposomes [191]. The lipid composition and surfactant concentration can be changed to offer long-term sustained medication delivery through the design of release kinetics for encapsulated medicines. This lowers the rate of medication distribution and increases patient compliance. For instance, Rahman et al. (2025) found that the controlled release of PTX from surfactant-stabilized liposomes improved antitumor activity in breast cancer models while lowering the negative effects usually associated with free PTX [192]. The clinical translation of surfactant-stabilized liposomes is beset with difficulties, including scaling up manufacturing and guaranteeing consistent drug release profiles, despite the potential benefits. However, these issues will be resolved by additional developments in liposome formulation and surfactant technology. Future research will concentrate on creating stimuli-responsive liposomes, adjusting surfactant–lipid ratios, and assessing long-term stability to guarantee that such systems may be successfully used in clinical breast cancer therapy [193].

### 4.4. Self-Emulsifying Drug Delivery Systems (SEDDSs)

Self-emulsifying drug delivery systems (SEDDSs) are being explored as an innovative surfactant-based solution for enhancing the bioavailability of drugs with poor water solubility. When SEDDSs are subjected to aqueous environments, such as those in the gastrointestinal tract, they spontaneously form microemulsions from an oil–surfactant–co-surfactant mixture. The drug disperses quickly as a consequence of this spontaneous formation, enhancing its solubility and absorption [194]. Improving the oral bioavailability of lipophilic drugs, like PTX and DOX, that are commonly administered to treat breast cancer is a significant advantage of SEDDSs [195]. A PTX SEDDS formulation significantly enhanced drug solubility and oral bioavailability in animal models, as reported in studies by Wahi et al. (2023), and it might serve as a substitute for intravenous drug delivery [196]. SEDDS formulations are especially useful for low-solubility drugs in aqueous systems because they can solubilize high concentrations of hydrophobic drug molecules. This is particularly important for the treatment of breast cancer because many potent chemotherapy drugs, such as paclitaxel, exhibit poor water solubility. According to Rathore et al. (2023), SEDDS formulations have the ability to enhance the solubility of such drugs, leading to increased plasma levels and improved therapeutic effects [197]. SEDDSs can also reduce drug crystallization, which prevents aggregates from presenting that would otherwise reduce drug bioavailability.

Another notable feature of SEDDSs are their capacity to improve medication penetration through biological membranes. Two of the surfactants employed in SEDDSs, Tween 80 and Cremophor EL, can improve membrane fluidity and decrease gut barrier or tumor cell membrane barrier characteristics [198]. According to a study by Lakkakula et al. (2024), PTX-loaded SEDDSs improved the drug’s permeability across the intestinal wall, leading to improved systemic absorption [199]. Likewise, SEDDS formulations of DOX have been shown to increase drug absorption in breast cancer cells, potentially improving therapeutic efficacy and lowering the likelihood of resistance [199]. It is also amazing how SEDDSs can overcome drug resistance. In the treatment of breast cancer, MDR, which is typically mediated by P-gp efflux pumps, can reduce the efficacy of chemotherapy medications like DOX. The ability of SEDDSs to block or avoid P-gp activity has been investigated in recent research [200,201]. SEDDS formulations of DOX improved intracellular drug absorption in MDR cell lines, according to Riaz et al. (2023), indicating that surfactants might affect P-gp activity and increase the lethal effects of chemotherapeutic medications [201].

Furthermore, SEDDSs can be customized to be administered orally and parenterally, allowing for greater flexibility in the course of treatment. The use of SEDDSs is a promising method for oral delivery of breast cancer treatment because of its capacity to encapsulate hydrophobic medications and protect them from deterioration while being transported through the gastrointestinal tract. As established by Wahi et al. (2023), the use of a lipid-based SEDDS formulation for oral administration of PTX not only increased drug solubility but also lowered the rate of administration compared to conventional PTX formulations, hence improving patient compliance [196]. Notwithstanding the benefits, there are still issues with the development of SEDDS for the treatment of breast cancer, namely with regard to formulation stability, scale-up procedures, and long-term shelf life. Future studies will concentrate on optimizing surfactant blends and enhancing the stability and controlled-release properties of SEDDS formulations in an attempt to enhance therapeutic effects [202]. All things considered, SEDDSs present a tenable strategy for improving the effectiveness of medications with low solubility in the treatment of breast cancer.

### 4.5. Biosurfactant-Based Nanocarriers

Natural amphiphilic molecules known as biosurfactants have attracted significant attention to drug delivery due to their low toxicity, biocompatibility, and biodegradability [203]. These surfactants, which are secreted by bacteria and fungus, have the ability to spontaneously form micelles and nanoparticles that can encapsulate a variety of therapeutic substances. Biosurfactant-derived nanocarriers present a promising approach to treating breast cancer since they are less immunogenic, more stable, and more drug-soluble than synthetic surfactants [204]. Studies by Ceresa et al. (2023) have demonstrated that rhamnolipid-derived micelles may encapsulate hydrophobic medications such as paclitaxel, providing a substitute for formulations that use synthetic surfactants [131]. One of the main advantages of biosurfactant-based nanocarriers is their biocompatibility, which allows for systemic circulation for extended periods of time without experiencing appreciable toxicity. According to a study by Wadhawan et al. (2022) [205], rhamnolipid micelles loaded with DOX demonstrated improved biocompatibility and preserved therapeutic efficacy in breast cancer cells with lower cytotoxicity than their synthetic equivalents. The fact that these natural surfactants are biodegradable, that is, the body will metabolize them without allowing them to build up in tissues, is a significant advantage over synthetic surfactants that may have harmful long-term effects.

In addition, more effective drug targeting and delivery could be achieved through biosurfactant-based nanocarriers. The cancer cell selectivity of the nanocarriers can be enhanced by modifying the biosurfactant micelle surface with targeting ligands such as folate or antibodies. For example, studies by Yoo and Park, 2004 showed that DOX can be delivered specifically to folate receptor-positive breast cancer cells through folate-conjugated rhamnolipid micelles, increasing treatment outcomes [206]. Biosurfactant-based systems are ideal candidates for precision therapy in the treatment of breast cancer because they are biocompatible and targetable. The ability of biosurfactants to modify biological processes, such as enhancing the absorption of drugs and bypassing multidrug resistance (MDR), has been explored in addition to their application in drug delivery. Naughton et al. (2019) state that biosurfactants like sophorolipids have been found to enhance intracellular drug delivery in drug-resistant cancer cell lines by blocking the P-gp efflux pump [207]. Biosurfactant-based nanocarriers have one of the most significant advantages of being able to reverse MDR, making them a preferred option for maximizing the efficacy of chemotherapy in the event of resistant breast cancer [207].

Moreover, biosurfactants have the ability to enhance nanoparticle stability and loading capacity due to their surfactant properties. By inhibiting nanocarriers from agglomeration, biosurfactants can enhance the stability of nanocarriers in biological media. Compared to traditional surfactant formulations, Bezza et al. (2020) demonstrated that biosurfactant-stabilized nanoparticles exhibited enhanced stability and longer release profiles [208]. Sequestering a high therapeutic load in stable nanocarriers is essential to make chemotherapy both effective and less harmful. The cost and large-scale production of biosurfactant-based nanocarriers pose challenges despite the promising prospects. Current research is focused, however, on scaling up biosurfactant production and optimizing the production processes. Biosurfactant-controlled drug delivery systems could be an effective tool for improving the outcomes of breast cancer treatment after these problems are addressed [209,210,211].

**Table 2:** Overview of nanoparticle/biosensor-based drug delivery systems in cancer therapy.

Type of Nanoparticle/Nanomaterial/Biosensor	Coated Materials	Description of the Study	Mechanistic Insights	Applications	Ref.
Lipid-based Nanoparticles	Lipid-based	Targeted lipid-based drug delivery systems for the treatment of lung cancer.	Presents the development of lipid nanoparticles to deliver drugs targeted at lung cancer.	Targeted drug delivery for the therapy of lung cancer.	[127]
Particulate Drug Delivery Systems	Nanoparticles, microparticles	Inhalable particulate systems for the treatment of lung cancer, with emphasis on nanoparticles, nanocomposites, and nanoaggregates.	Examines the effectiveness of inhaled particulate delivery systems for treating lung cancer.	Lung cancer therapy, drug delivery.	[128]
Nanoemulsions	Various surfactants	Nanoemulsions as carriers for the treatment of lung cancer—investigating their stability and drug loading.	Comprehensive assessment of the characteristics of nanoemulsions in lung cancer treatment.	Lung cancer therapy, drug delivery.	[129]
Copper Nanoparticles	Surfactant-coated	Synthesis, antimicrobial activity, and nucleic acid binding of a copper (II) complex with phenanthroline.	The research investigates the antimicrobial activity and nucleic acid interaction.	Antimicrobial, anticancer uses.	[130]
Biosurfactants	N/A	Utilization of biosurfactants for biomedical and pharmaceutical applications.	Investigates the biomedical applications of biosurfactants.	Drug delivery, applications in the pharmaceutical industry using biosurfactants.	[131]
Micelles-based Systems	Various polymers	Micelle-based systems for different industrial applications.	Explains the drug delivery versatility of micelles and their uses in industry.	Drug delivery, diverse industrial applications.	[132]
Polymeric Micelles	Various polymers	Polymeric mixed micelles for enhancing anticancer drug delivery.	Investigates the role of polymeric micelles in increasing drug efficacy.	Anticancer drug delivery.	[136]
Polymeric Micelles	Various polymers	Polymeric micelles for the treatment of breast cancer: progress, clinical translations, and regulatory issues.	Focuses on the clinical translation of polymeric micelles for breast cancer treatment.	Breast cancer treatment, drug delivery.	[137]
Polymeric Nanocarriers	Polymeric materials	Polymeric nanocarriers for cancer treatment.	Investigates the use of polymeric nanocarriers for the treatment of cancer.	Cancer therapy.	[138]
Cobalt (III) Complexes	Surfactant-coated	Investigation of cobalt (III) complexes and their binding with nucleic acids and biological systems.	Investigates how cobalt complexes coated with surfactants react with DNA/RNA and have antimicrobial activity.	Drug delivery, antimicrobial uses.	[139]
Polymeric Micelles	Pluronic P123/F127	Co-delivery of doxorubicin and paclitaxel by pluronic micelles for multidrug-resistant tumors.	Investigates how pluronic micelles reverse drug resistance in tumors.	Cancer treatment, reversal of multidrug resistance.	[146]
Polymeric Micelles	Various polymers	Polymeric micelles for multidrug delivery in cancer treatment.	Investigates the co-delivery of drugs using polymeric micelles.	Cancer treatment, drug delivery.	[147]
Nanoparticles	N/A	Nanoemulsions to enhance the anticancer activity of paclitaxel.	Studies the improvement in drug accumulation and efficacy using nanoemulsions.	Cancer treatment, delivery of paclitaxel.	[142]
Niosomes	Non-ionic surfactants	Niosomes as a drug delivery system targeted for the treatment of breast cancer.	Focus on the targeted delivery of drugs to breast cancer cells using niosomes.	Breast cancer treatment, drug delivery.	[192]
Polymeric Micelles	Pluronic-based	Polymeric micelles for breast cancer targeted drug delivery.	Studies the use of polymeric micelles for enhancing drug delivery in breast cancer therapy.	Breast cancer treatment.	[166]
Green Synthesis Nanoparticles	Eosin-Y	Green synthesis of silver nanoparticles for selective L-Dopa detection.	The study emphasizes green chemistry for synthesizing nanoparticles for sensitive detection.	Biomedical applications, detection systems.	[167]
Silver Nanoparticles	N/A	Green synthesis and biological activity of silver nanoparticles for anticancer and antimicrobial activity.	Investigates silver nanoparticles’ roles in anticancer activity, antimicrobial effects, and DNA cleavage.	Anticancer, antimicrobial uses.	[178]

## 5. FDA-Approved or Clinical Stage Surfactant-Containing Formulations

Surfactant-based drug delivery systems enhance drug solubility, stability, and targeting, and thus therapeutic effects. A number of such systems are approved by the FDA, whereas others are being tested in the clinical setting for treating cancer (Table 3 and Table 4). These systems help to evade drug resistance and optimize treatment. Many formulations with surfactants have successfully received FDA approval or clinical use, which shows the promise of surfactants in enhancing drug delivery for various diseases, including cancer. Doxil (liposomal doxorubicin), a surfactant-stabilized liposomal formulation, especially with polyethyleneglycol (PEG), is one of the best-documented examples [212,213,214,215,216,217]. Through the application of the enhanced permeability and retention (EPR) effect to target tumor tissues more effectively, Doxil, an FDA-approved drug, significantly improves the pharmacokinetics of doxorubicin, enhancing its solubility and reducing systemic toxicity [218]. When surfactants are used to prepare liposomes, hydrophobic drugs can be encapsulated for controlled and sustained release, which improves the efficacy of cancer therapy [219]. Abraxane (albumin-bound paclitaxel), another FDA-approved, surfactant-containing formulation, employs albumin as a stabilizing molecule in a surfactant-free formulation. The formulation confers a significant advantage in treating non-small-cell lung cancer and breast cancer by enhancing the solubility of paclitaxel and enabling its delivery to tumor cells through receptor-mediated endocytosis [220,221].

Clinical trials are also testing formulations based on surfactants to give new treatments. For example, clinical-stage lipid-based formulations based on surfactants, like Marqibo (liposomal vincristine sulfate), are being studied for the treatment of lymphoma, leukemia, and other cancers [222]. The formulation enhances vincristine’s pharmacokinetics by decreasing its quick clearance and neurotoxic adverse effects [223,224]. Furthermore, a variety of micelles and surfactant-containing nanoemulsions are undergoing clinical testing to improve the bioavailability and delivery targeting of medications that are poorly soluble in water, such as curcumin and paclitaxel [225,226,227]. Nanoemulsion-based formulations, like those employed in the Cemiplimab clinical study, are being investigated to more effectively deliver therapeutic proteins and small molecules to target tissues, in addition to improving drug stability and circulation time [228,229,230,231,232,233,234]. Clinical-stage and FDA-approved formulations demonstrate the important role surfactants play in enhancing drug distribution and getting around the drawbacks of conventional chemotherapy [38,39]. Surfactants help solubilize hydrophobic medications, shield them from degradation, and allow for targeted administration to particular sites, all of which increase efficacy and decrease toxicity [40]. Furthermore, surfactant-containing systems are useful because they may encapsulate a variety of drugs, such as proteins, nucleic acids, and tiny molecules, expanding their use in the treatment of numerous malignancies and other illnesses [235]. The ongoing development and improvement of these formulations may advance pharmaceutical administration procedures and offer patients safer and more efficient treatment options.

### 5.1. Genexol^®^ (Polymeric Micelle Formulation of Paclitaxel)

Genexol^®^ is a polymeric micelle formulation of paclitaxel designed to overcome the drawbacks of conventional paclitaxel formulations [236]. This formulation increases paclitaxel’s aqueous solubility and stability by entrapping it in polyethyleneglycol (PEG)-poly(D,L-lactide) block copolymers [237]. Genexol^®^ (Samyang Biopharmaceuticals Corporation, Seoul, South Korea) was designed to enhance the therapeutic efficacy of paclitaxel and reduce its toxicities, particularly when used to treat ovarian and breast cancers [238]. Paclitaxel’s half-life is prolonged and its bioavailability is enhanced by the formulation of micelles, which prevents the drug from degrading too rapidly in the blood [239]. The micellar nanoparticles are small enough to allow the increased permeability and retention (EPR) effect, which enhances cancer cell targeting and allows selective tumor tissue accumulation [240].

Genexol^®^ has demonstrated encouraging outcomes in a number of clinical trials. In a Phase III trial of patients with metastatic breast cancer, Genexol^®^ was just as effective as traditional paclitaxel, with a marked decrease in hypersensitivity responses and neutropenia [240]. Additionally, Genexol^®^ showed a better pharmacokinetic profile than traditional paclitaxel, which resulted in fewer adverse effects and greater tolerance [241]. Nevertheless, despite its benefits, Genexol^®^ has drawbacks. The formulation’s long-term stability, especially under different storage conditions, may have an impact on the stability of its therapeutic effects, which is one of the primary issues [242]. Furthermore, the PEG micelle component may cause immunological side effects in specific patient populations, which could affect the drug’s long-term use [243].

Clinical trials have investigated the possibility of combining Genexol^®^ with different anticancer medications [244]. When used in combination with cisplatin or trastuzumab, for instance, Genexol^®^ has been demonstrated to increase the cytotoxicity of paclitaxel and improve overall treatment results in a variety of solid tumors [245]. In an effort to enhance cancer treatment plans and enhance patient outcomes, clinical trials of these combinations are still being investigated. Future clinical trials will concentrate on improving micelle formulation in order to address stability concerns and enhance patient outcomes for patients with various cancer types. The creation of dual-drug-loaded micelles is one method for co-administering paclitaxel with other targeted or chemotherapeutic medications in a single micellar platform; this could potentially have a synergistic effect [246].

### 5.2. Emerging TPGS-Based Micellar Drugs in Trials

Tocopheryl polyethylene glycol succinate (TPGS)-based micelles have proven to be a promising method for addressing hydrophobic drugs’ solubility problem in cancer treatment [247,248,249]. Vitamin E derivative TPGS acts as a surfactant to enhance the solubility, bioavailability, and systemic toxicity of poorly soluble drugs [250]. Preclinical and clinical investigations have assessed TPGS-based micelles as drug delivery formulations of a variety of drugs, including paclitaxel and other anticancer therapeutics [251]. Micelles formed by TPGS exploit TPGS’s ability to solubilize hydrophobic drugs but maintain their stability in aqueous environments. The micelles also allow release of the drug within the target location and enhance cellular uptake by endocytosis [252]. By promoting selective accumulation in cancer tissues through the EPR effect, the application of TPGS in drug delivery systems enhances the therapeutic efficacy of chemotherapy [253].

Numerous preclinical investigations have shown that TPGS-based micelles can reduce adverse effects and increase the effectiveness of chemotherapeutic medications, for example, TPGS micelles have been successfully used to encapsulate chemotherapeutic medications such as doxorubicin and paclitaxel, and these micelles have demonstrated enhanced anticancer efficacy [254,255,256]. By increasing drug solubility and lowering the necessary dosages, these medication formulations improve patient compliance and lower toxicities. Clinical trials have shown encouraging safety and efficacy outcomes for formulations based on TPGS. For instance, clinical research using paclitaxel-loaded TPGS micelles showed better pharmacokinetic profiles, including improved tumor-targeting capabilities and less severe side effects such as hypersensitivity reactions, when compared to the conventional paclitaxel formulation [257,258].

**Figure 3:**
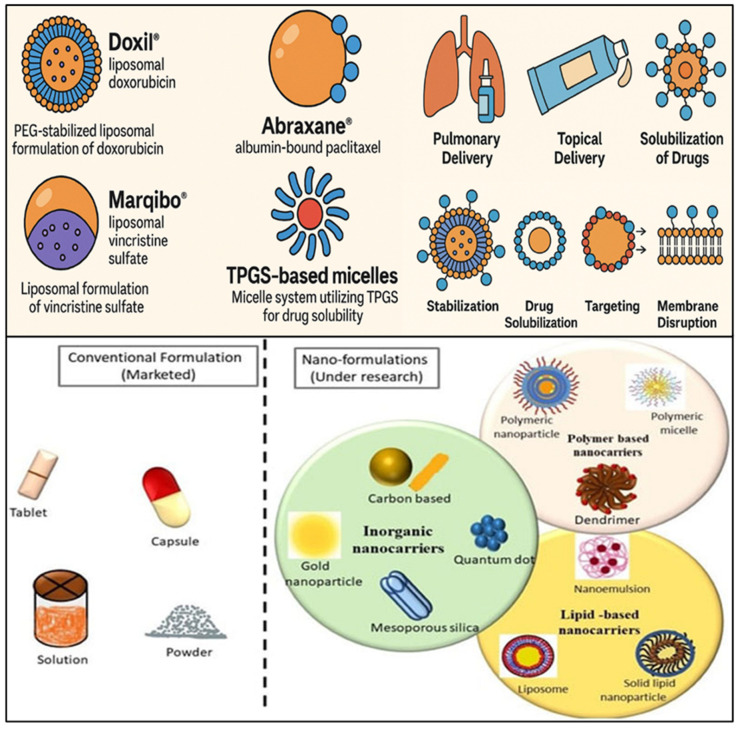
FDA clinical-stage surfactant-containing nano formulations.

Despite the advantages, TPGS-based micellar formulations have problems when used in clinical settings. One of the main worries with TPGS is its possible immunogenicity, especially since cancer patients are often given it [259]. Furthermore, when TPGS-based micelles are manufactured at scale, consistency and cost issues could arise, which would prevent their widespread application in clinical settings [260]. The main goal of the current research on TPGS-based micelles is to improve the drug release profiles and targeting properties. Nanotechnology advancements like stimuli-responsive drug release systems are being used in TPGS-based formulations to distribute drugs in a regulated and targeted manner in response to environmental stimuli like pH or temperature [261]. The development of TPGS-based micelles may improve the therapeutic results of cancer treatment and lessen its adverse effects. FDA clinical trials for surfactant-based nanomedicines are highlighted in Figure 3. In the treatment of cancer, the products improve drug targeting, solubility, and bioavailability. They aim to address the issues of drug resistance and improve the efficacy of treatment.

TPGS-based micelles, which are prepared with D-α-tocopheryl polyethylene glycol succinate (TPGS), have gained considerable interest because they have the ability to enhance drug solubility and bioavailability. Their ability to bring about immunogenicity, however, is a major deterrent to their use in the clinic. Recent data have proved that PEGylated molecules like TPGS have the ability to generate anti-PEG antibodies, resulting in accelerated blood clearance (ABC) and hypersensitivity reactions on repeated dosing [253,254,255]. The amphiphilic character of TPGS is also a concern with regard to complement activation-related pseudoallergy (CARPA), particularly at high doses or chronic dosing. Although TPGS has been found to possess an acceptable safety profile in preclinical studies, comprehensive immunotoxicity evaluations are only limited, and additional studies are required to prove safety in a broad spectrum of patient populations. From a production standpoint, large-scale production of TPGS-micelles is also hampered by batch-to-batch reproducibility issues, particularly with respect to particle size, drug loading efficiency, and stability [256]. Micellization is highly sensitive to the solvent system, concentration, and preparation procedure, and scale-up is certainly no easy process [257,258]. Furthermore, regulatory avenues are uncertain, considering that TPGS is both an active ingredient with an effect on pharmacokinetics and a functional excipient. Such dual functionality makes classification based on current FDA or EMA guidelines challenging, and the outcome is that it might have to be characterized and controlled with more stringent standards than typical excipients. Harmonized nanomedicine approval guidelines are also not in place, further complicating the picture and potentially slowing clinical deployment of TPGS–micellar systems in the face of promising therapeutic outcomes [261,262].

**Table 3:** Surfactant-based drug delivery formulations (FDA-approved and clinical-stage).

Formulation	Type	Surfactant/Carrier	Drug	Indications	Key Advantages	Status	Ref.
Doxil^®^ (Janssen Biotech, Horsham, PA, USA)	PEGylated liposome	PEG (polyethylene glycol)	Doxorubicin	Ovarian cancer, Kaposi’s sarcoma, multiple myeloma	Enhanced EPR effect, reduced systemic toxicity, prolonged circulation	FDA Approved	[218]
Marqibo^®^	Liposomal	Lipids + surfactants	Vincristine sulfate	Acute lymphoblastic leukemia	Prolonged circulation, reduced neurotoxicity	FDA Approved	[216]
Genexol^®^-PM	Polymeric micelles	PEG-PLA (block copolymer surfactant)	Paclitaxel	Breast, ovarian, NSCLC	No Cremophor EL, reduced toxicity, improved stability and tumor accumulation	Approved in Korea, Clinical Trials Elsewhere	[238,241,245]
TPGS–Paclitaxel	Micellar formulation	TPGS (d-α-Tocopheryl polyethylene glycol)	Paclitaxel	Various solid tumors (ongoing trials)	Improved solubility, reduced hypersensitivity, enhanced tumor targeting via EPR	Clinical Trials	[225,229,232,234]
TPGS–Doxorubicin	Micellar formulation	TPGS	Doxorubicin	Breast, liver cancer (preclinical/clinical)	Enhanced uptake, lower dose requirement, reduced toxicity	Clinical Trials	[206,218]
Curcumin Nanoemulsion	Nanoemulsion	Surfactants (e.g., Tween 80, lecithin)	Curcumin	Breast, prostate, pancreatic cancer (under study)	Enhanced oral bioavailability, better stability, improved targeting	Clinical Trials	[225]
Cemiplimab Nanoemulsion	Nanoemulsion	Surfactant-based emulsion system	Cemiplimab (monoclonal antibody)	Cutaneous squamous cell carcinoma (trial stage)	Enhanced targeting, extended circulation, improved protein delivery	Clinical Trials	[226]
Micelle-Cisplatin/Trastuzumab Combo	Polymeric micelle combo therapy	PEG-PLA or TPGS systems	Cisplatin + Trastuzumab	HER2+ breast and other solid tumors	Synergistic targeting, improved bioavailability, dual-drug delivery potential	Clinical Research	[40]

**Table 4:** Surfactant-based nanomedicines in breast cancer: clinical trials, approvals, and advances.

Study	Findings	Breast Cancer Nanomedicine	Ref.
Evolution of surfactant therapy for respiratory distress syndrome: past, present, and future	Historical overview of surfactant-based respiratory therapies	Basis for understanding surfactant mechanisms and evolution to nanomedicine	[212]
Surfactant systems: their use in drug delivery	Traditional review of surfactants in drug delivery systems	Early basis for today’s surfactant-based nanocarriers	[213]
Surfactants, nanomedicines and nanocarriers: a critical evaluation on clinical trials	Evaluation of surfactants in nanomedicine clinical trials	Emphasizes clinical translation potential of surfactant-based nanocarriers	[214]
VSLI (Marqibo^®^): phase I study in children and young adults	Clinical trial of liposomal vincristine	Demonstrates lipid-based delivery in oncology	[216]
Review of FDA-approved lipid-based drugs	Analysis of FDA-approved lipid-based products	Regulatory and translational observations	[217]
30 years of Doxil^®^: updated analysis	Review of PEGylated liposomal doxorubicin	Primary model for targeted nanomedicine in breast cancer	[218]
Lipid-based nanocarriers for breast cancer	Review of lipidic systems in BC treatment	Long discussion of surfactant-involving carriers in BC	[220]
Solid lipid nanoparticles and NLCs	Progress in lipid nanocarriers for BC	Explains surfactant role in stabilizing SLNs/NLCs	[221]
Cremophor EL: drawbacks & advantages	Comprehensive analysis of surfactant Cremophor^®^ (BASF Corporation, Florham Park, NJ, USA) EL	Emphasizes the issue of surfactant toxicity	[231]
Dendrimer and micelle formulations	Overview of micellar systems for BC	Surfactant role in micelle-based delivery	[40]
Glucose-targeted mixed micelles outperform Genexol	Mixed micellar delivery system improves BC therapy	Example of targeted surfactant-stabilized system	[245]
Functionalized polymeric micelles	From design to clinical trials in cancer	Surfactant-stabilized micelles for targeting	[246]
TPGS-based nanomedicine	Vitamin E TPGS in drug delivery and theranostics	TPGS as surfactant and MDR-reversal agent in BC	[249]
Cetuximab-conjugated TPGS micelles	Delivery of docetaxel for TNBC	TPGS micelles as multifunctional nanoplatforms	[256]
Halofuginone in TPGS micelles for TNBC	TPGS micelles enhance efficacy in TNBC	Exhibits targeted delivery and MDR reversal	[262]

## 6. Targeted Delivery Approaches

**Figure 4:**
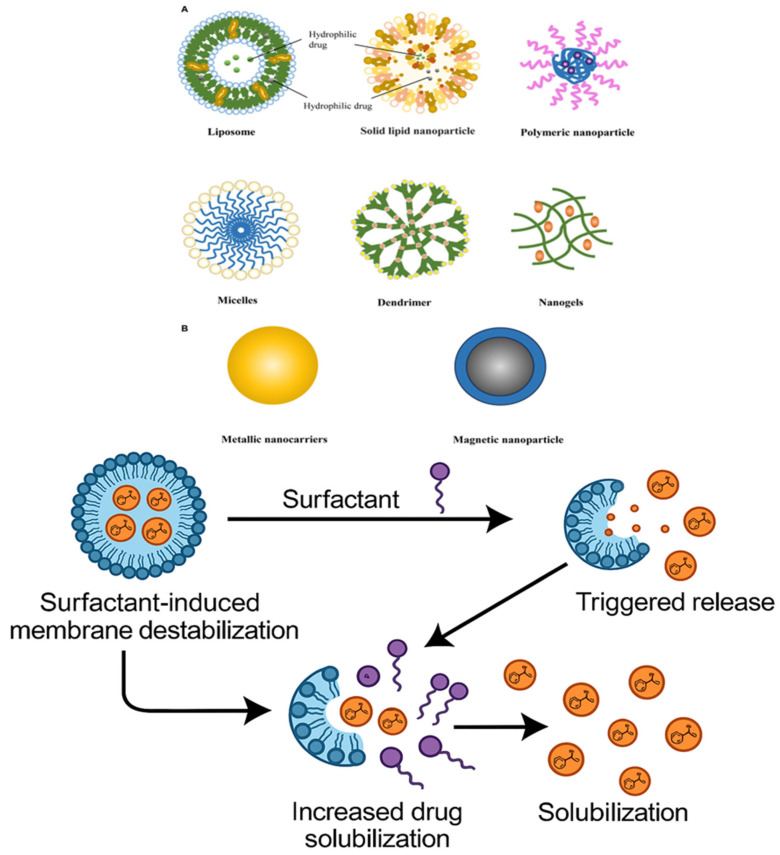
Surfactant-assisted drug-release mechanisms from nanocarrier systems. (**A**) Liposome and micelles; (**B**) metallic nanocarriers and magnetic nanoparticle.

Surfactant-mediated drug-releasing strategies from nanocarrier systems encompass enhancing drug solubility, modifying cellular membranes, and inhibiting efflux pumps. Surfactants stabilize nanocarriers, facilitate drug loading, and allow for controlled or site-specific release. This approach enhances therapy efficacy, especially for multidrug-resistant tumors (Figure 4). To achieve the highest therapeutic effects and minimize systemic side effects, targeted drug-delivery systems are designed to direct therapeutic molecules to specific cells or tissues. In oncology, where conventional chemotherapy often leads to severe side effects due to the nonspecific distribution of drugs in the body, the ability to deliver drug action to a particular location is incredibly beneficial [263]. Targeted drug delivery has been achieved through many strategies, including the application of liposomes, nanoparticles, and antibody–drug conjugates, each having shown potential in preclinical and clinical studies [264]. These systems usually make use of the biological properties of tumors, such as their increased permeability and retention (EPR) effect, to allow for improved drug accumulation in the tumor region [265]. One of the most popular methods for delivering drugs to a specific location is through surface-modified nanoparticles. For instance, nanoparticles containing specific ligands or antibodies may bind to overexpressed receptors on tumor cells to enable the exact administration of the therapeutic ingredient [266]. This method increases the drug’s effectiveness while lowering the risk of systemic toxicity. In the well-known instance of using HER2-targeted liposomes to deliver chemotherapeutic medicines for the treatment of breast cancer, trastuzumab coupled to the liposomal surface enhanced drug delivery and clinical results [267].

The regulated release of drugs is another benefit of targeted delivery systems based on nanoparticles. When encapsulated in nanocarriers, which may release the drug in response to particular stimuli like temperature or pH changes frequently found in the tumor microenvironment, drugs can be protected from premature degradation [268]. These methods make it possible to apply the medication more effectively, which improves therapeutic results and lessens adverse effects. Paclitaxel, for instance, can be given in pH-sensitive nanoparticles, which introduce the medication into the acidic tumor environment and increases the drug’s lethality against cancer cells [269]. A further promising method for targeted drug delivery is the use of antibody–drug conjugates, or ADCs. ADCs, which are composed of monoclonal antibodies associated with these medications, enable the selective delivery of potent cytotoxic agents to cancer cells exhibiting the particular target antigen [270]. This strategy has already produced FDA-approved treatments for HER2-positive breast cancer, such as trastuzumab emtansine (Kadcyla^®^ (Roche, Basel, Switzerland)) [271]. ADCs’ high levels of specificity reduce the collateral harm that traditional chemotherapy usually causes to healthy cells and tissues.

Although targeted medication delivery systems have advanced significantly, there are still a number of obstacles to overcome. One of the biggest obstacles is tumor heterogeneity, which makes it more difficult to successfully target every cancer cell because various parts of a tumor express varying amounts of the target receptor [272]. Furthermore, the immune reaction to the antibodies or nanoparticles may cause the drug delivery system to be eliminated before it reaches the target, which would decrease the treatment’s overall efficacy [273]. Research continues to develop ways in which one can bypass such limitations, such as developing delivery systems that are multi-targeted or utilizing stealth technology in order to evade immune detection [274]. All things considered, targeted drug delivery techniques have transformed the science of drug therapy as a whole by providing better and more accurate ways to deliver medications to their intended locations of action. Clinical treatments based on liposomes, ADCs, and nanoparticles have demonstrated significant promise in lowering adverse effects and improving therapeutic results. More research and clinical trials are required to improve these systems and address the ongoing issues with their use, which will eventually result in even more potent treatments for cancer and other diseases.

### 6.1. Ligand-Functionalized Surfactant-Based Systems (e.g., Folic Acid, HER2 Antibodies)

Drug delivery via ligand-functionalized surfactants has emerged as an effective strategy to enhance the efficacy and selectivity of targeted therapy. Such systems involve the encapsulation of pharmaceutical compounds with surfactants, which are amphiphilic molecules capable of forming lipid layers or micelles. The systems have the ability to transport drugs to cells overexpressing specific receptors, like HER2, by incorporating ligands such as folic acid or antibodies against these receptors. This enhances treatment effectiveness and reduces off-target toxicity [275]. Improved treatment results have been obtained when therapeutic drugs are concentrated in the targeted tissues, such as cancer cells, due to the targeting capability provided by ligands such as folic acid, which can bind with the folate receptor, or HER2 antibodies, which target HER2-positive cells specifically [276]. Since folate receptors are broadly overexpressed on a range of cancer cell types, such as breast, ovarian, and colon cancers, folic acid-targeting surfactant-based systems are particularly useful in cancer therapy [277]. Such systems are able to utilize the increased folate uptake of the tumor to deliver chemotherapeutic drugs, such as doxorubicin (DOX) or paclitaxel, to the tumor site directly by conjugating surfactant-based carriers with folic acid [278]. As per research, this approach can raise the level of medication that builds up in tumor tissues, enhancing the therapeutic index and reducing the adverse effects that are normally related to conventional chemotherapy [279].

Likewise, HER2-targeted surfactant-based delivery systems have shown tremendous potential in the therapy of HER2-positive cancers, such as certain types of gastric and breast cancer. Such drug delivery systems have the ability to bind selectively to HER2 receptors overexpressed on the surface of cancer cells by functionalizing surfactants with antibodies against HER2 [280]. This allows the encapsulated drug to be released predominantly at the site of the tumor [281]. This tailored strategy improves clinical outcomes for HER2-positive tumors by minimizing damage to healthy cells and optimizing the therapeutic efficacy of the encapsulated medication, such as doxorubicin or trastuzumab [282]. Ligand-functionalized surfactant-based systems provide improved drug targeting, stability, and controlled release characteristics. Depending on the tumor microenvironment’s acidic or enzymatic conditions, the encapsulated drug can be administered, and surfactant micelles or vesicles can shield it from early deterioration while in circulation [283]. This regulated release technique lessens systemic side effects and increases the treatment’s therapeutic efficacy by guaranteeing that the medication is exclusively active at the intended site [284].

One of the ways that ligand-targeting and surfactant-based delivery systems can be used to introduce several therapeutic treatments is by combining chemotherapy with gene therapy or immunotherapy. For instance, surfactant-based carriers functionalized with folic acid or HER2 antibodies have been used to co-deliver chemotherapy drugs and siRNA, enabling synergistic effects across several therapeutic modalities [285]. This approach facilitates the use of combination medications, which can circumvent drug resistance and enhance the overall therapeutic response [286]. Although ligand-functionalized surfactant-based systems have a lot of potential, several issues must be resolved to maximize their practical usefulness. These include concerns about the ligands’ immunogenicity, the stability of the ligand–receptor interaction, and the requirement for effective production procedures. Furthermore, intratumoral variability in target receptor expression, such as HER2, limits the therapeutic potential of targeted medications in certain patient groups [287,288]. It will need more research and clinical testing to overcome these obstacles and fully utilize ligand-functionalized surfactant-based drug delivery systems.

### 6.2. Dual-Delivery Systems (e.g., DOX + siRNA)

Dual-delivery platforms are a sophisticated method of drug delivery that has attracted a lot of attention lately. They involve the simultaneous co-delivery of multiple medicinal agents. Conjugating nucleic acids like siRNA with traditional chemotherapeutic treatments like doxorubicin (DOX) can greatly increase the therapeutic effectiveness of anticancer medications [287,288,289,290,291,292,293,294,295,296,297,298,299,300,301]. By intercalating DNA and blocking topoisomerase II, DOX is a potent chemotherapeutic drug that causes DNA damage and apoptosis in tumor cells [302]. However, its usefulness is frequently restricted by severe systemic toxicity and drug resistance. However, siRNA offers an alternative mode of action that can get around these restrictions by mutating particular genes linked to drug resistance or the development of cancer. The goal of designing a dual-delivery system is to concurrently target a few pathways that give cancer cells their ability to survive. For instance, co-delivery of DOX and siRNA against multidrug resistance (MDR) proteins, such as P-glycoprotein (P-gp), has been demonstrated to sensitize drug-resistant cancer cells to DOX treatment, hence overcoming drug resistance and improving overall therapeutic efficiency [303]. Dual-delivery systems, in contrast to traditional medications, can produce synergistic benefits by transporting both therapeutic substances via a single delivery method. This leads to better medication results and fewer adverse effects [304].

Because they provide a consistent and regulated release platform for DOX and siRNA, nanoparticles are essential in the administration of dual therapy. To guarantee their simultaneous release at the tumor site, DOX and siRNA have been encapsulated in various nanoparticles, including liposomes, micelles, and polymeric nanoparticles [305,306,307,308,309,310,311]. The nanoparticles may be surface-functionalized with certain ligands or antibodies, such as folic acid or HER2-targeted antibodies, to improve targeting efficacy and increase drug accumulation within the tumor environment [312]. This focused strategy is especially helpful in resolving the problem of non-specific distribution, which frequently results in off-target toxicities and reduced effectiveness of traditional drug delivery methods. The potential for synergistic effects is one of the main benefits of dual-delivery methods. For example, siRNA can be used to decrease genes that encourage treatment resistance, whereas DOX directly affects the cancer cells. This combination can increase the effectiveness of chemotherapeutic medications against cancer cells and offer a more effective long-term treatment plan by decreasing tumor recurrence [313]. Research has demonstrated that co-administration of DOX with siRNA-targeting genes such as drug efflux pumps or anti-apoptotic proteins dramatically improves response to treatment in a variety of cancer models [314].

Another significant benefit of dual-delivery systems is the controlled release of both drug substances. A more efficient and sustained therapeutic effect can be achieved through the design of nanoparticles that simultaneously release siRNA and DOX [315]. For instance, the use of pH-responsive nanoparticles enables the regulated administration of siRNA and DOX in the acidic environment of tumors, guaranteeing that both medications reach the tumor cells at the best possible moment [316]. By lowering the possibility of early release and the resulting harm to healthy tissues, this improves the treatment’s overall safety profile. Despite their potential, clinical translation and the creation of dual-delivery systems present difficulties. Among the primary problems are the intricate formulation and the challenge of attaining consistent and reliable co-delivery of both therapeutic medications. Furthermore, resolving siRNA’s intrinsic instability and guaranteeing the effective cellular uptake of siRNA and DOX continue to be formidable obstacles [317]. Future studies must concentrate on enhancing these systems’ stability, target efficacy, and controlled release behavior in order to optimize their therapeutic potential.

## 7. Overcoming Multidrug Resistance (MDR)

**Figure 5:**
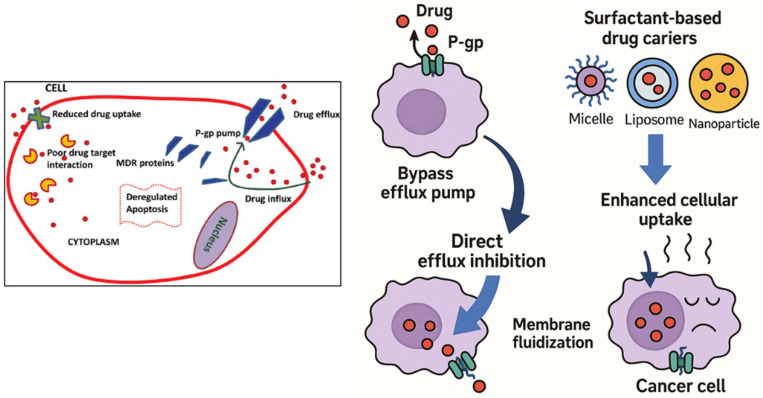
Mechanisms of MDR reversal using surfactant-carriers.

Surfactant-carriers suppress drug efflux pumps such as P-glycoprotein and increase membrane permeability. Surfactant-carriers enhance intracellular drug uptake and stabilize drug delivery systems (Figure 5). This results in re-established drug sensitivity and enhanced cancer treatment efficacy. In the treatment of breast cancer, surfactant-based nanocarriers enhance drug solubility, stability, and targeted delivery. Surfactant-carriers enhance intracellular drug retention and counteract multidrug resistance (MDR) by inhibiting efflux pumps such as P-glycoprotein (Table 5 and Table 6). These technologies minimize toxicity and side effects as well as enhance therapeutic outcomes. Multidrug resistance (MDR) is the biggest hindrance in cancer treatment, making most chemotherapeutic treatments fail. MDR is mainly due to overexpression of efflux pumps such as P-glycoprotein (P-gp), which actively exports anticancer drugs from cells, restricting drug buildup and effectiveness [318,319,320,321,322,323,324]. This can be further enhanced by alterations in drug targets, modified apoptosis mechanisms, and increased DNA repair processes [325,326,327]. Scientists have been focusing on developing methods to either inhibit these efflux pumps or increase drug delivery to bypass these resistance processes to combat MDR. One approach is the employment of drug delivery systems using nanoparticles, which are able to encapsulate drugs and deliver them directly to the site of the tumor [328,329]. This increases the deposition of drugs within cancer cells and reduces their binding to efflux pumps in normal tissues by increasing the solubility and bioavailability of anticancer drugs; nanoparticles like liposomes [330], micelles, and polymeric nanoparticles have shown promise against MDR. Through the modulation of the release of their payload, such nanoparticles are able to ensure long-term drug concentrations within the tumor environment and reduce the risk of efflux pump-dependent resistance [331,332]. As an illustration, evidence has been put forward to the effect that the delivery of liposomal formulations of doxorubicin is capable of circumventing P-gp-driven efflux through the encapsulation of the drug in a lipid bilayer that prevents direct drug interaction with the pump and increases intracellular drug accumulation [333].

Apart from enhancing drug delivery, the inhibition of P-gp and other drug efflux pumps is another essential approach to overcome MDR. A number of compounds have been found to inhibit the functioning of these pumps, thus reversing the resistance of cancer cells to chemotherapy. In drug-resistant cancer cell lines, for example, it has been found that the calcium channel antagonist verapamil is an inhibitor of P-gp activity and enhances the cytotoxicity of drugs like doxorubicin [348,349]. More recent studies have also explored the use of naturally occurring compounds like curcumin that have the capacity to modulate the expression and activity of efflux pumps and small molecules that specifically inhibit P-gp, such as tariquidar and zosuquidar [350]. While concerns regarding their safety and toxicity remain to be dealt with in the clinical context, these inhibitors can effectively bypass drug resistance when administered in combination with conventional chemotherapy [351]. Utilizing multidrug resistance modulators, which have the ability to alter gene expression associated with resistance in cancer cells, is another interesting approach. The downregulation of P-gp or other resistance-associated protein expression by gene therapy methods is one such case. To revive the sensitivity of cancer cells to chemotherapeutic drugs, P-gp and other drug resistance-related genes have been accurately knocked down by employing RNA interference (RNAi) and CRISPR-Cas9 gene editing [352]. It is possible to enhance the effectiveness of existing drugs without having to develop new chemotherapeutic drugs by using these technologies to specifically target the molecular pathways responsible for MDR [353].

To overcome MDR, combined therapy that hits multiple resistance pathways has also been proposed. Scientists attempt to render cancer cells more responsive to treatment and less prone to developing resistance by combining chemotherapeutic agents with inhibitors of resistance pathways, i.e., P-gp inhibitors or apoptosis modulators. For example, preclinical models of MDR cancer have shown enhanced antitumor activity when doxorubicin is used in combination with a P-gp inhibitor or an apoptosis inducer [354]. Clinical trials are ongoing to study these combinatory strategies, with the aim of finding feasible methods to overcome MDR and improve the efficacy of cancer therapy. While promising developments have been seen in combating MDR, there remain some challenges to be addressed before these strategies can be applied in the clinical context. Major hurdles involve the potential toxicity of resistance inhibitors, complexity of resistance mechanisms, and diversity of malignancies [355]. In addition, to ensure efficacy and safety, extensive preclinical and clinical studies are required to develop novel drug delivery strategies and resistance modulators. In search of optimal approaches to reverse MDR and maximize the efficacy of cancer therapies in the clinic, continued investigation is needed.

### 7.1. Role of Surfactants Like TPGS, Tween 80, and Brij 35 in Efflux Pump Inhibition

One major challenge in cancer therapy is multidrug resistance (MDR), where cancer cells are resistant to numerous chemotherapy agents. MDR is primarily brought about by efflux transporters, particularly the ATP-binding cassette (ABC) family of transporters, which export anticancer drugs from cells, reducing their intracellular levels and efficacy [335,356,357]. Surfactants like Tween 80, Brij 35, and d-alpha-tocopheryl polyethylene glycol 1000 succinate (TPGS) have been recognized as potential efflux pump inhibitors (EPIs) that could assist in reversing MDR. The capability of TPGS, a derivative of vitamin E, to inhibit P-glycoprotein (P-gp), a recognized efflux pump, is established. TPGS has been shown in studies to reverse drug resistance by enhancing the intracellular concentration of chemotherapeutic drugs such as doxorubicin and paclitaxel by inhibiting the ATPase activity of P-gp [337,343]. Similarly, it has been proven that the nonionic surfactant Tween 80 suppresses P-gp-efflux in a variety of cell lines, enhancing the efficacy of chemotherapeutic drugs [344]. Experiments have indicated that another nonionic surfactant, Brij 35, may enhance the cytotoxicity of anticancer drugs in drug-resistant cell lines by modifying the activity of efflux pumps [338].

The physicochemical properties of the surfactants, e.g., hydrophilic-lipophilic balance (HLB), are also the reasons for the ability of surfactants to inhibit efflux pumps. For example, TPGS may interact with the cell membrane lipid bilayer and change P-gp activity due to its high HLB. Besides enhancing drug uptake, such interaction prevents drugs from actively effluxing out of the cell [358]. The ability of surfactants to alter the fluidity of the membrane and inhibit the efflux pump is crucial for enhancing medication retention in cells. Nonionic surfactants like Tween 80 and Brij 35 are also more desirable for clinical application because they are less toxic to healthy cells compared to other efflux pump inhibitors [359,360]. Their capability to bypass chemoresistance is also evidenced through their differential effect on MDR cells [361]. In addition, clinical trials have indicated promising results when surfactants and anticancer drugs are co-administered. For example, the ability of TPGS and paclitaxel in combination to overcome P-gpmediated drug resistance in cancer cells has been explored in clinical situations [336]. As per clinical trials using surfactant–drug formulations, surfactants have the ability to enhance the bioavailability and therapeutic effects of the medications substantially. Surfactants such as TPGS possess beneficial pharmacokinetic properties enabling them to escape efflux-mediated resistance and thus are an effective strategy in combating MDR in anticancer therapy [362,363]. Drug-resistant cancer patients can be improved with the treatment if surfactants are incorporated into drug formulations.

### 7.2. Mechanisms of MDR Reversal with Surfactant-Carriers

**Figure 6:**
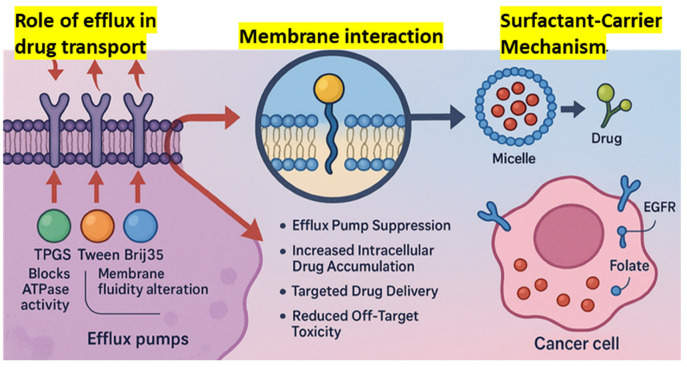
Overcoming multidrug resistance with surfactant-assisted drug delivery.

Surfactants increase drug solubility, block efflux pumps, and raise membrane permeability. They enable greater intracellular drug retention in MDR cells. This approach enhances therapeutic effectiveness and helps counteract multidrug resistance (Figure 6). Surfactant-carriers are increasingly being utilized as a strategy to overcome MDR by not only blocking efflux pumps but also by increasing the bioavailability and cellular accumulation of chemotherapeutic agents [364,365]. Surfactant interaction with cell membranes is the process through which surfactant-carriers induce reversal of MDR [366,367]. Surfactants, being amphiphilic, are able to dismantle the plasma membrane lipid bilayer, facilitating increased drug molecule permeability that otherwise would be pumped out by pumps like P-gp. The increased membrane permeability facilitates chemotherapy drugs to accumulate within the cells, which ensures drug retention and bypasses mechanisms of resistance [368]. Surfactants also have the role of being solubilizing agents, which enhance the solubility of poorly soluble anticancer drugs and facilitate easier transport across membranes [339].

Another reason why surfactants and chemotherapeutic drugs act together is the formation of surfactant–drug complexes, which are more readily taken up by cancer cells than free drugs. Increased intracellular drug levels may be attained through encapsulating hydrophobic drugs within micellar assemblies that bypass the efflux pump barriers [345]. When surfactant and drug molecules combine to create mixed micelles, hydrophobic drugs are stabilized and made more soluble, enhancing their prospects for the treatment of MDR-resistant cancers [346]. In addition, by altering the transcriptional activity of efflux pump protein-encoding genes or by influencing the efflux pump proteins themselves, surfactants are able to alter efflux pump protein expression levels, leading to a reduction in their activity and enhanced retention of drugs [369]. Surfactant-carriers can interfere with other cellular mechanisms that have been involved in drug resistance in addition to their role in suppressing the efflux pump. For instance, it has been shown that surfactant use influences signaling pathways that are also connected with MDR, including those that involve breast cancer resistance protein (BCRP) and multidrug resistance proteins (MRPs). Surfactants have the ability to increase the reversal of MDR by specifically targeting these proteins [340]. Surfactant carriers can also affect intracellular drug distribution and can be targeted at various subcellular compartments, including the nucleus, where most anticancer drugs act, and make the pharmaceuticals more evenly distributed within the cells. This comprehensive approach reduces the challenges posed by MDR and enhances the overall efficacy of the treatment regimen [370].

Surfactants’ use as carriers has also promoted the development of targeted drug delivery systems aimed at specifically tackling MDR. The occurrence of off-target effects can be reduced by targeting the drug–surfactant complex to specific sites of tumors through the modification of surfactant molecules to incorporate targeting ligands, i.e., peptides or antibodies. Besides enhancing the tumor drug concentration, these surfactant-carrier targeting systems decrease the systemic toxicity associated with conventional chemotherapy [341]. Raising the therapeutic index of drug delivery systems based on surfactants has been shown to be feasible by targeting specific receptors that are overexpressed on cancer cells, i.e., folate receptors or epidermal growth factor receptors (EGFRs) [347]. This customized drug delivery system holds much potential for conquering MDR and enhancing the selectivity and effectiveness of chemotherapeutic drugs. Overall, because surfactant-carriers can specifically target some cancer cells, increase drug solubility and stability, and inhibit efflux pumps, they represent a convenient means of combating MDR. Surfactants in combination with chemotherapeutic medicines might enhance the effectiveness of cancer therapy, particularly in the ability to overcome resistance. For them to maximize their potential in reversing MDR and improving patient outcomes in cancer therapy, additional research into surfactant formulation optimization and clinical validation are needed [342].

## 8. Biocompatibility and Toxicological Profile

When developing surfactant-based drug delivery systems, biocompatibility and toxicological characteristics are crucial (Table 7), particularly when surfactants are being utilized to treat cancer and combat multidrug resistance (MDR). Because surfactants like TPGS, Tween 80, and Brij 35 are frequently given systemically, biocompatibility is crucial to proving their safety in vivo. According to studies, TPGS has been extensively investigated for its high biocompatibility and low toxicity, and it is said to be well tolerated in animal models [371]. Since nonionic surfactants Brij 35 and Tween 80 have shown reduced cytotoxicity in a number of in vitro and in vivo investigations, they are both viable options for drug administration [372,373]. Notwithstanding this possibility, the toxicity profiles of any drug can be altered by dosage and administration methods. Among the negative effects caused by extremely high concentrations or extended exposure to these surfactants are hemolysis and inflammation, which emphasizes the necessity of optimizing formulation conditions in order to reduce toxicity [374]. Furthermore, it is clear that large dosages of surfactants, such as Tween 80, may be harmful to kidney and liver function; therefore, careful dosing control is necessary to prevent systemic toxicity [375]. Therefore, assessing organ-specific toxicities as well as acute and chronic toxicity is necessary to ensure the safe and efficient clinical use of surfactant carrier systems. Lastly, despite the promising biocompatibility profiles of surfactants like TPGS, Tween 80, and Brij 35, more toxicological testing is necessary to guarantee their safety for use in overcoming MDR, and comprehensive research is necessary to establish long-term safety within human clinical trials.

### 8.1. Cytotoxicity Studies and Dose-Limiting Toxicities

Cytotoxicity studies are a crucial component of assessing the safety and effectiveness of surfactant-based drug delivery systems, particularly when surfactants are employed in anticancer therapy to combat multidrug resistance (MDR) [376]. Determining the possible negative effects of surfactants on healthy cells and tissues is the aim of these investigations. In several cell lines, the cytotoxicity profiles of surfactants such TPGS, Tween 80, and Brij 35 have been identified. Research has indicated that TPGS dosages up to 0.5% (*w*/*v*) are well tolerated in in vitro cell models and have demonstrated encouraging outcomes in improving drug retention at therapeutic levels without significantly harming normal cells [377]. However, it should be mentioned that elevated surfactant levels may result in dose-dependent cytotoxicity. For example, it has been shown that elevated Tween 80 levels can have cytotoxic effects, such as cell membrane rupture and changes in cell viability [378]. To prevent exacerbating the toxicity to healthy tissues, surfactants and chemotherapeutic drugs may also have additive or synergistic effects that need to be carefully considered [379]. Using their understanding of cytotoxicity profiles, scientists can tailor surfactant formulation and dosage to maximize therapeutic efficacy while reducing negative effects on healthy cells.

The development of medication delivery methods that include surfactants raises serious concerns regarding DLTs, which are side effects that, because of their severity, make it impossible to increase a drug’s dosage further and may even cause treatment to be stopped. In surfactant–drug combos, DLTs may result from the combined toxicity of the surfactant and the chemotherapeutic medication. For instance, while TPGS has been demonstrated to increase the bioavailability of doxorubicin and paclitaxel, excessive dosages of the drug have been linked in animal models to hepatotoxicity, gastrointestinal toxicity, and blood enzyme rise [380]. Likewise, overdosing on Tween 80 has been linked to nephrotoxicity and liver damage [381]. These DLTs highlight how crucial it is to identify the ideal dosage range for drug–surfactant mixes in order to reduce adverse effects. Clinically investigated surfactant-based drug delivery systems require close patient monitoring for toxicity, especially when bigger dosages or longer treatment are administered. Preclinical research is frequently used to determine safe and effective dosages, and additional surfactant formulation adjustments can lower DLTs by lowering toxicity without compromising therapeutic efficacy [382].

### 8.2. Biodegradable and GRAS-Listed Surfactants

Biodegradable surfactants have attracted the attention of drug delivery system researchers, especially for use in the treatment of MDR in cancer care [383]. Surfactants that break down into harmless metabolites are desirable since they lower the likelihood of long-term body accumulation. Due to their biocompatibility and biodegradability, poly(lactic acid)-derived biodegradable surfactants, such as poly(lactic-co-glycolic acid) (PLGA), have been used in diverse drug delivery formulations [384]. Even though PLGA-based surfactants degrade over time, no possibility of bioaccumulation or toxicity exists for this formulation. They are ideal for sustained drug delivery systems due to their biodegradability, which can release the drug slowly over time without requiring additional treatments. In addition, by promoting more drug retention in cancer cells and less exposure to healthy cells, these surfactants can be designed so as to target a specific tissue, enhancing their value in breaking MDR [385]. As a result, biodegradable surfactants are suitable candidates for safer and more effective MDR reversal methods with their controlled release properties and biocompatibility.

Whether a surfactant is included by regulatory organizations such as the U.S. Food and Drug Administration (FDA) as Generally Recognized as Safe (GRAS) is another factor in deciding which one to use in delivering medication. Surfactants which have been scientifically proven to be safe for their intended application in food, drugs, or cosmetics are known as GRAS-listed surfactants. Due to their safety profile, nonionic surfactants such as Tween 80 and GRAS-listed surfactants are often used in drugs [386]. At proper amounts, Tween 80 has been extensively researched for its lack of toxicity and potential to enhance the solubility and bioavailability of drugs that poorly dissolve without causing significant adverse effects [387]. Brij 35 is another GRAS-listed nonionic surfactant, which has exhibited negligible cytotoxicity when subjected to in vitro and in vivo experiments. Its applications in enhancing therapeutic effect and overcoming MDR without inducing side effects have also motivated investigations on its application in drug delivery systems [388]. GRAS-listed surfactants are suitable for clinical translation and long-term therapy because they provide an added layer of safety and regulatory assurance.

Because they meet the sustainability and safety standards needed for widespread use in medical applications, GRAS-listed and biodegradable surfactants are particularly sought-after [389]. Because they cannot build up in the body, their capacity to break down into non-toxic byproducts lowers the possibility of harmful long-term effects, including organ toxicity or chronic inflammation [390]. Furthermore, the minimal environmental impact of surfactants due to natural degradation is one of the main elements driving the pharmaceutical industry’s increased desire for green chemistry solutions. Because they can significantly improve the hydrophobic medication’s solubility, stability, and controlled release in drug delivery systems, surfactants are especially useful in combating MDR [391,392].

### 8.3. Biodegradability and the Environmental Impact of Surfactants

Although biodegradable surfactants provide several advantages in medication administration, it is important to take the environment into account as well. The overall sustainability and safety of biodegradable surfactants may even be impacted by their environmental persistence. Certain biodegradable surfactants, such those derived from natural oils and fatty acids, have been demonstrated to break down more quickly and produce less harmful consequences than synthetic surfactants [393,394]. However, the rate at which certain surfactants biodegrade can be affected by environmental variables like pH, temperature, and microbial activity. It is essential to assess the in vivo and environmental degradation profiles of surfactants used in drug administration to make sure they do not present long-term risks to ecosystems. Ongoing research on surfactant biodegradation pathways and environmental toxicity will be crucial to the development of sustainable medication delivery methods with fewer negative effects on biology and the environment [395].

Using GRAS-listed and biodegradable surfactants in drug delivery formulations is one method of avoiding MDR while upholding strict safety regulations. Clinical usage of surfactants such as TPGS, Tween 80, and Brij 35 is possible due to their low toxicity and good tolerance at therapeutic concentrations [396]. More research is required to evaluate their cytotoxicity, dose-limiting toxicities, and biodegradation characteristics in preclinical and clinical contexts. The creation of safer surfactant–drug formulations will require striking the ideal balance between maximizing therapeutic efficacy and reducing toxicity to healthy tissues. By concentrating on biodegradable and ecologically friendly surfactants, scientists can create cancer therapies and develop other therapeutic areas that are more effective and sustainable while also increasing the overall efficacy of MDR reversal methods [397,398].

**Table 7:** Overview of surfactants in drug delivery, with focus on mechanisms, applications, and safety profiles.

Type of Surfactant	Coated Materials	Description of the Study	Mechanistic Insights	Applications	Ref.
TPGS (α-Tocopheryl polyethylene glycol 1000 succinate)	Various drug molecules (e.g., doxorubicin, paclitaxel)	Studied for improving drug bioavailability and overcoming MDR; demonstrated good in vivo biocompatibility and low toxicity.	Inhibits P-glycoprotein (P-gp) efflux, enhances drug retention in tumor cells, low cytotoxicity at ≤0.5% *w*/*v*.	Cancer therapy, MDR reversal	[371,377,380]
Tween 80 (Polysorbate 80)	Chemotherapeutics and nanoparticle surfaces	Evaluated for systemic use in drug delivery; noted reduced cytotoxicity but dose-dependent toxicity at high concentrations.	Enhances solubility and cellular uptake; excessive doses linked to hepatotoxicity and nephrotoxicity.	Drug solubilization, cancer drug delivery	[372,375,378,381]
Brij 35 (Polyoxyethylene lauryl ether)	Nanocarriers and hydrophobic drugs	Investigated for improving solubility and overcoming MDR with minimal cytotoxicity observed in studies.	Improves membrane permeability, synergistic effects with chemotherapeutics; low toxicity at therapeutic doses.	Drug delivery systems, MDR treatment	[373,379,388]
PLGA-based biodegradable surfactants	Nanoparticles and encapsulated drugs	Biodegradable surfactant used for sustained drug release, reducing long-term accumulation risks.	Controlled drug release, degradation into non-toxic metabolites, improved targeting to tumor cells.	Sustained-release systems, cancer therapy	[384,385]
Natural oil and fatty acid-derived surfactants	Eco-friendly drug carriers	Evaluated for biodegradability and reduced environmental impact; faster breakdown into harmless byproducts.	Biodegradable, environmentally friendly, supports green pharmaceutical development.	Sustainable drug delivery	[393,394]
GRAS-listed surfactants (e.g., Tween 80, Brij 35)	Pharmaceutical formulations	Certified safe for use in drugs, cosmetics, and food by the FDA; extensive biocompatibility profiles.	Low toxicity at proper dosages, improves bioavailability without significant side effects.	Clinical drug delivery formulations	[386,387,388]

## 9. Recent Trends and Innovations in Stimuli-Responsive Surfactant Systems

**Figure 7:**
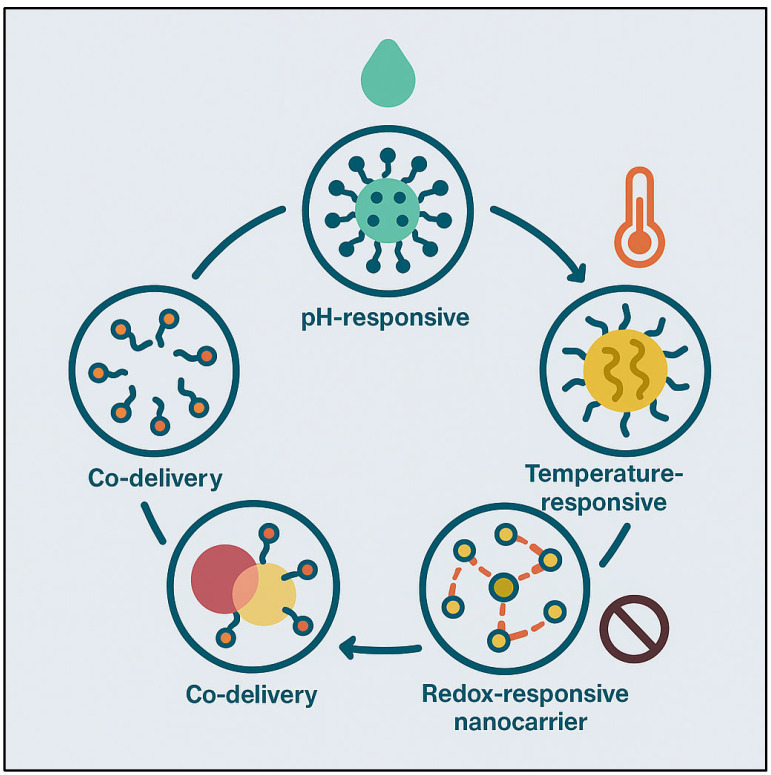
Stimuli-sensitive surfactants that alter their properties in response to pH, temperature, or redox conditions.

Stimuli-sensitive surfactants or smart surfactants have recently been a breakthrough family of materials (Figure 7) due to their ability to alter structural and physicochemical properties upon exposure to external environmental signals such as pH, temperature, and redox [399,400,401,402]. It is possible to design surfactants that can selectively respond to specific environmental stimuli [403,404], and hence, these surfactants are suitable for various biomedical and industrial applications (Table 8). Recent work has pointed to the development of surfactants with pH-sensitive moieties, which are extremely useful for drug delivery systems in cancer chemotherapy [403,404]. Ding et al. (2022) reported that pH-sensitive surfactants may deliver chemotherapeutic agents into acidic tumor microenvironments with lower pH values than normal tissue [405]. In addition, temperature-sensitive surfactants, which can exhibit phase transitions at certain temperatures, have been of interest in controlled drug release systems. Verkhovskii et al. (2023) found that such surfactants are applied in smart hydrogels for localized and temperature-induced drug release, enhancing the therapeutic effect [406].

Redox-responsive surfactants, however, are finding increased use in those applications wherein the release of bioactive chemicals is facilitated by reduction or oxidation of certain chemical bonds [407]. Disulfide-containing surfactants, for example, have been utilized to design nanoparticles that, upon contact with the reducing microenvironment of tumor cells or inflammatory tissue, release their payload [408]. Shende and Deshpande, 2020 assert that by minimizing off-target effects and enhancing the therapeutic index, redox-responsive surfactant systems may enhance drug delivery’s specificity and efficacy [409]. Targeting hard-to-reach locations such as the brain or inflamed tissues is easier with this targeted release, triggered by changes in redox conditions. In addition, surfactant systems’ responsiveness to numerous stimuli, such as pH and redox, offers the opportunity to maximize drug release processes, broadening their application in precision medicine.

### 9.1. Co-Delivery of Chemotherapeutics and Immune Agents

The concurrent delivery of chemotherapeutic drugs and immune-modulatory drugs has received considerable interest in cancer therapy with the synergistic benefits it has to provide [410]. Combining immunotherapies, which stimulate the immune system of the body against tumors, with chemotherapy, which specifically kills the cancer cells, is a promising method to circumvent the limitations of single-agent monotherapy [411]. Surfactants are very important to this co-delivery approach, particularly in the development of nanocarriers capable of simultaneously encapsulating both types of drugs. Kandasamy et al.’s study in 2023 demonstrated that surfactant-stabilized micelles could co-deliver a checkpoint inhibitor and the chemotherapy agent doxorubicin, which enhanced tumor regression and reduced systemic toxicity [412]. Successful delivery of immunological and chemotherapeutic drugs to the tumor site relies on the surfactant’s ability to solubilize hydrophobic drugs and protect them from premature degradation. The use of surfactant systems for co-delivery may not only enhance drug efficacy but also overcome the problem of tumor immune suppression. For instance, surfactant-based nanoparticles may enhance the overall treatment outcome by encapsulating immune stimulators and chemotherapeutics in one formulation, such as cytokines or adjuvants. Liu et al. (2024) explored the use of surfactant-functionalized lipid nanoparticles to co-deliver paclitaxel and an immune checkpoint inhibitor [413]. The results showed a significant increase in immune cell activation at the tumor site along with enhanced tumor-targeted delivery. The tumor microenvironment was effectively transformed by this bimodal approach, which additionally directly cytotoxically destroyed the cancer cells and induced an anti-tumor immune response. Through synergistic co-delivery, these findings demonstrate the value of surfactant-based nanocarriers in revolutionizing cancer treatment.

### 9.2. Surfactants in Photodynamic and Gene Therapy

Two newer therapeutic methods being explored increasingly for their ability to target and treat tumors selectively with minimal side effects are photodynamic therapy (PDT) and gene therapy [414,415]. Surfactants have been found to be key players in both PDT and gene therapy as they can facilitate the solubilization and controlled release of therapeutic agents like photosensitizers and nucleic acids. The stability and bioavailability of photosensitizers that are photoactivated to produce reactive oxygen species (ROS) targeted at killing cancer cells are enhanced in PDT through the use of surfactants. Recent advances have focused on making the selectivity and efficacy of PDT better through surfactant-coated nanoparticles that can efficiently deliver these photosensitizers to tumor cells [416,417]. For instance, Castillo et al. (2017) reported that upon exposure to light activation, surfactant-stabilized micelles of Porphyrin-based photosensitizer exhibited improved cellular uptake and retention with more efficacious therapeutic outcomes [418].

Surfactants are utilized in gene therapy to assist in delivering genetic material into target cells, such as plasmids, siRNAs, and CRISPR-Cas9 reagents [419]. Cationic-headed surfactants are often utilized to co-associate with nucleic acids to form nanoparticles that may penetrate cell membranes. The use of surfactant-based nanoparticles [420] for the delivery of CRISPR-Cas9 systems for gene editing within cancer cells was highlighted in a recent study by Wu et al. (2024). By protecting the cargo from enzymatic degradation [420,421], the surfactants enhanced gene editing efficacy and enhanced the stability and cellular internalization of the CRISPR reagents. In addition, surfactant-based systems may be designed to respond to specific stimuli, for example, temperature or pH, in a controlled manner to deliver their gene therapy payloads. These developments render surfactant-based carriers a fundamental tool in the delivery of efficient and targeted PDT and gene therapy strategies.

### 9.3. Surfactant-Mediated Tumor Targeting

Surfactant-based systems’ capacity to obtain selective tumor targeting is a decisive aspect of their success as drug delivery systems [421]. Leverage on some characteristics of tumor cells, including their altered surface charge, receptor overexpression, and the unique characteristics of the vasculature of tumors, is involved in the multifaceted procedure of tumor targeting [422]. Surfactant systems may be engineered to enhance tumor passive and active targeting, as recent studies indicate. To deposit the therapeutic payload precisely in the location of the tumor, for example, surfactant-modified nanoparticles may be ligand-functionalized and attach to receptors overexpressed on the surface of tumor cells [423]. As per a Pisani, 2022 study, folic acid-coated surfactant-functionalized liposomes selectively targeted cancer cells that had the expression of the folate receptor, resulting in greater drug concentration in tumors [424]. The enhanced permeability and retention (EPR) effect, which allows nanoparticles to accumulate within tumor tissues due to their leaky vasculature, can also be exploited by surfactant-based systems as well as receptor-mediated targeting [425]. By improving the stability and size distribution of nanoparticles, surfactants can increase their extravasation into tumors while decreasing absorption by healthy tissues. Recent advancements in surfactant formulations have produced hybrid nanoparticles, which mix liposomes, micelles, and dendrimers for the best possible drug delivery. According to research by Lee et al., these surfactant hybrids improve the pharmacokinetic profile of the encapsulated medications and improve tumor-specific delivery, resulting in more effective treatment with fewer systemic side effects [426,427].

Despite their promise for cutting-edge therapeutic applications, surfactant-based systems’ clinical translation and development continue to face numerous obstacles [428]. The requirement for exact control over the physicochemical characteristics of surfactant formulations in order to guarantee constant performance is one of the main obstacles. Understanding surfactants’ interactions with biological systems, such as the immune system, cytotoxicity, and biocompatibility, is crucial to maximizing their safety and effectiveness [429]. Scalability and reproducibility are also essential for the commercialization of surfactant-based nanocarriers, which are sometimes hindered by intricate and expensive production procedures [430]. Researchers like Chen et al. (2023) have emphasized the significance of creating standardized protocols in surfactant formulation in order to address these issues and enhance consistency in treatment results [431].

The development of surfactant systems that can guarantee site-specific and regulated drug release in vivo is another crucial problem. Although stimuli-responsive surfactants have demonstrated potential for initiating medication release under specific circumstances, it is still challenging to guarantee the exact moment and site of release. Future research will probably concentrate on increasing the sensitivity of surfactant systems to several stimuli at once, such as pH, temperature, and redox conditions, in order to accomplish more dependable and effective drug delivery [432]. As the sector develops, it is anticipated that new surfactant-based materials with enhanced targeting efficiency, stability, and therapeutic efficacy will be created.

**Table 8:** Recent advances in stimuli-responsive surfactant systems for targeted drug delivery and therapeutic applications.

Surfactant	Coated Materials	Description of the Study	Mechanistic Insights	Applications	Ref.
pH-responsive surfactants	Chemotherapeutic-loaded carriers	Ding et al. (2022) created pH-sensitive surfactants for drug delivery in acidic tumor microenvironments [405].	pH-induced structural transitions release the drug in acidic tumor microenvironment.	Targeted drug delivery in cancer therapy	[405]
Temperature-responsive surfactants	Hydrogels	Verkhovskii et al. (2023) employed temperature-sensitive surfactants in smart hydrogels to allow localized drug release [406].	Heat-induced phase transition induces drug release.	Controlled drug delivery systems	[406]
Redox-responsive surfactants	Disulfide bond-containing nanoparticles	Shende and Deshpande (2020) investigated redox-responsive systems for targeted drug release in tumors [409].	Redox environment (e.g., tumor cells) breaks disulfide bonds, releasing drugs.	Targeted drug delivery with enhanced specificity	[409]
Dual pH- and redox-responsive surfactants	Multifunctional nanoparticles	Mixed pH/redox stimuli-responsive systems discussed to maximize drug release mechanisms [407].	Stimuli-induced controlled release, responsive to tumor microenvironment.	Precision medicine and targeted therapies	[407,408]
Surfactant-stabilized micelles	Co-delivery nanocarriers (doxorubicin + checkpoint inhibitor)	Kandasamy et al. (2023) investigated micelles for the concurrent delivery of chemo- and immunotherapy agents [412].	Surfactant micelles solubilize hydrophobic drugs, safeguarding them until tumor site arrival.	Cancer co-therapy (chemo + immunotherapy)	[412]
Surfactant-functionalized lipid nanoparticles	Lipid-based co-delivery vehicles (paclitaxel + immune agent)	Liu et al. (2024) showed lipid nanoparticles to promote immune activation and tumor targeting [413].	Enhanced immune cell activation and targeted drug delivery by nanoparticle encapsulation.	Synergistic cancer immunochemotherapy	[413]
Surfactant-stabilized micelles	Porphyrin-based photosensitizers	Castillo et al. (2017) indicated improved PDT efficacy through surfactant micelles.	Enhanced photosensitizer delivery, cellular uptake, and ROS generation on light activation.	Photodynamic therapy (PDT)	[418]
Surfactant-coated nanoparticles	Photosensitizer-loaded particles	Studies [418] highlighted improved ROS production for targeted killing of cancer cells during PDT [416,417].	Tumor selectivity and light-induced activation.	Photodynamic cancer therapy	[416,417]
Cationic-headed surfactants	CRISPR-Cas9 delivery nanoparticles	Wu et al. (2024) used surfactant nanoparticles for the delivery of gene-editing cargo [420].	Surfactants protect and stabilize nucleic acids, increasing gene-editing efficiency.	Gene therapy (CRISPR delivery)	[420]
Surfactant-functionalized liposomes	Folic acid-decorated liposomes	Pisani (2022) developed folate-targeted liposomes for targeted tumor drug delivery [424].	Targeted receptor-mediated cell uptake by folate receptor-overexpressing cancer cells.	Tumor-specific drug targeting	[424]
Surfactant-based nanoparticles	EPR-effect exploiting systems	Nanoparticles leverage tumor vasculature leakiness to increase accumulation [426].	Passive tumor targeting by size and stability modulation.	Cancer drug delivery	[426]
Hybrid surfactant systems	Liposome-micelle-dendrimer hybrids	Lee et al. (2022) designed hybrid surfactant systems with enhanced delivery and pharmacokinetics [427].	Synergistic combination of multiple nanocarriers for improved drug delivery and lowered toxicity.	Advanced cancer therapeutics	[426,427]
Standardized surfactant formulations	Various drug delivery systems	Chen et al. (2023) stressed scalable and reproducible surfactant manufacture [431].	Providing uniform drug release behavior and biocompatibility.	Clinical translation of nanocarriers	[431]
Multi-stimuli-responsive surfactants	Smart nanoparticles	Recent progress covered the design of surfactants that respond to pH, redox, and temperature in combination [432].	Multifunctional responsiveness for extremely specific drug release.	Next-generation targeted therapies	[432]

## 10. Challenges and Future Prospects in Surfactant Metal Complexes for Biomedical Applications

### 10.1. Scale-Up, Reproducibility, and Storage Stability

The development of surfactant–metal complex systems for biomedical applications is significantly hampered by the difficulty of scaling up and repeating their synthesis. Although these complexes can be successfully synthesized in a lab setting, a number of obstacles must be removed before they can be produced commercially. Nanoparticles must have regulated size, shape, and composition in order to be successful, especially in medication delivery systems. Asymmetric treatment results and toxicity issues may result from variations in these factors. Temperature, reaction duration, and solvent selection are among of the variables that can affect the synthesis process’s repeatability throughout scaling [433,434,435]. Furthermore, storage stability is a major concern because many surfactant–metal complexes can degrade over time, particularly when exposed to environmental factors such humidity, light, and temperature variations [436]. Important steps to improve the long-term stability of such complexes include the creation of protective coatings or more sophisticated formulations that inhibit oxidation or hydrolysis and may reduce their bioactivity [87,90].

### 10.2. Regulatory Aspects

Surfactant–metal complexes used in biological applications have a complex and developing legal framework. These materials, which are frequently regarded as a novel type of nanomaterials, must pass stringent testing and authorization procedures before they can be widely used in therapeutic settings. Regulatory agencies like the European Medicines Agency (EMA) and the U.S. Food and Drug Administration (FDA) have developed frameworks for evaluating nanomaterials, but the regulations pertaining to surfactant-metal complexes are still being developed [437]. Toxicity tests, such as genotoxicity, immunotoxicity, and acute and chronic toxicity, should be performed to evaluate the safety of these complexes. Their biocompatibility with human tissue should also be thoroughly examined. Concerns regarding the consistency of their safety and effectiveness as well as regulatory obstacles are exacerbated by the diversity in surfactant–metal complex production. As research advances, a more uniform method of generating and evaluating these complexes will be needed to satisfy regulatory standards [438].

### 10.3. Precision Medicine and AI-Assisted Surfactant System Design

The development of surfactant–metal complexes for precision medicine holds great promise for the use of artificial intelligence (AI). Artificial intelligence (AI) has the potential to optimize these systems by searching through large datasets to find the best surfactants, metal pairings, and nanoparticle formulations for specific patients [439]. Additionally, scientists can use machine learning techniques to forecast the interactions between biological systems and surfactant–metal complexes, which can improve the effectiveness of treatments for particular disorders [440]. By taking patient genetic and phenotypic variations into account, AI can further assist in customizing drug delivery systems, guaranteeing that surfactant–metal complexes are made for maximum efficacy and minimal adverse effects [441]. AI-based models can also help predict the stability, biodegradation, and bioavailability of these complexes, ensuring their long-term efficacy in medical applications [442,443,444,445]. However, the creation of AI-supported systems must be carried out carefully since accurate model training necessitates both big datasets and high-quality experimental information.

There are still significant concerns about the possible toxicity and biocompatibility of surfactant–metal complexes, despite their great potential for biomedical applications. The way that nanoparticles interact with biological systems is greatly influenced by their size and surface chemistry. Stabilizing surfactants for metal nanoparticles might occasionally trigger immunological reactions that result in inflammation or cytotoxicity [446,447,448]. Furthermore, the metal ions that the nanoparticles release have the ability to build up in tissues and have toxicological effects, such as organ damage and oxidative stress [444]. Biocompatibility testing is essential to guarantee that the surfactant–metal complexes do not cause negative reactions when given to people. Future studies should concentrate on creating more biocompatible surfactants or altering the metal complexes’ surface characteristics in order to reduce toxicity while preserving therapeutic efficacy. Longer-term in vivo studies are also necessary to examine the potential accumulation of metal complexes in organs and tissues, which could eventually pose a health risk [449,450].

The transition of surfactant–metal complexes from laboratory-scale synthesis to clinical settings presents a second significant obstacle. Although these complexes appear promising in vitro, they frequently behave differently in vivo because of factors like metabolism, clearance rates, and biodistribution [445]. Manufacturing must be scalable without sacrificing the surfactant–metal complex’s structural integrity or functionality in order to advance these systems to clinical trials. Additionally, large-scale production methods need to be economical and ecologically responsible. Utilizing green chemistry concepts, such as bio-inspired or low-toxic reagents, during the synthesis of surfactant–metal complexes can lower manufacturing costs and their environmental impact [87,88,89,90]. Large-scale production involves taking into account the production and regulatory limitations, for example, complying with good manufacturing practices (GMPs). Ensuring that such products can be mass-produced without compromising their safety and quality is critical as scientists strive to optimize the synthesis and formulation process.

Looking forward, continued interdisciplinary research is crucial to the future of surfactant–metal complexes in biomedical applications. To surpass the current challenges and achieve the full potential of these materials, collaboration among chemists, biologists, and engineers will be important. Accelerated exploration of new surfactant–metal complex combinations will be facilitated by new material synthesis methods, such as automated synthesis platforms and high-throughput screening. In addition, there can be synergistic benefits to integrating nanomedicine with advanced technologies such as gene therapy, immunotherapy, and regenerative medicine. The future development of surfactant-assisted nanocarriers in the treatment of breast cancer will revolutionize the accuracy, efficacy, and safety of treatment. Surfactants play a pivotal role in stabilizing nanocarrier systems, improving drug solubility, and targeting drugs to tumor cells with minimal off-target toxicity [450]. Future development will focus on the development of smart, stimulus-sensitive surfactants for controlled drug release against the tumor microenvironment’s specific conditions, i.e., pH or enzyme levels. Additionally, the use of biocompatible and biodegradable surfactants will widen the clinical utility of these nanocarriers by reducing systemic toxicity and immunogenicity [451]. Future research can also explore the co-encapsulation of therapeutic drugs and diagnostic probes in a single nanocarrier for real-time monitoring and targeted therapy. With ongoing advancements in nanotechnology and molecular biology, surfactant-assisted nanocarriers are set to become a keystone in the next generation of breast cancer treatments. To enhance the precision and specificity of genetic treatments, surfactant–metal complexes, for instance, might be used as drug delivery vehicles for gene-editing tools. The development and optimization of surfactant–metal complexes for specific biological functions will increasingly rely on AI and computational modeling methods as they evolve. A promising future for the development of highly personalized and targeted therapies is offered by the integration of AI, precision medicine, and nanotechnology.

## 11. Conclusions

Surfactant-based drug delivery systems are a novel approach to breast cancer treatment that address some of the fundamental limitations of traditional chemotherapeutic drugs. Surfactants are amphiphilic, which allows them to act as permeability enhancers, solubilizers, and MDR modulators, all of which are especially important in the treatment of aggressive and drug-resistant breast cancer. They are effective vehicles in modern oncological treatment because they can produce nanoscale carriers, protect labile medicines, and promote tumor deposition through passive and active targeting methods. Clinical translation has demonstrated the safety and therapeutic efficacy of surfactant-containing formulations, as proven by medications like Genexol^®^, with successful clinical results. Recent precision medicine objectives are closely matched with improvements in ligand-mediated targeting and dual-delivery techniques, which further improve therapy precision and treatment synergy. Furthermore, surfactants’ functional profiles are being expanded through their incorporation into modern technologies such as gene therapy, phototherapy, and stimuli-responsive systems. Nonetheless, there remain significant barriers to overcome before they can reach their full clinical potential. These include pharmacokinetic predictability, long-term storage stability, production scalability, and regulatory framework uniformity. Because of the cytotoxicity of certain synthetic surfactants, safer, biodegradable alternatives must be developed and approved by regulators. Advances in biosurfactant technology and computational design tools such as machine learning and AI-guided formulation hold the key to overcoming these impediments. In short, drug delivery methods that are surfactant-based have evolved into a robust and incredibly versatile platform for the treatment of breast cancer. These technologies improve treatment outcomes through the integration of nanotechnology, pharmacokinetics, and tailored medicine. They also usher in a new age of cancer therapies that will be characterized by enhanced patient compliance, reduced systemic toxicity, and improved selectivity.

## Figures and Tables

**Table 5:** Surfactant-based nanoplatforms in breast cancer therapy and MDR reversal.

Nanoplatform/Strategy	Role of Surfactant		Targeting/MDR Mechanism	Key Outcomes	Ref. No.
Polymeric NPs, micelles, dendrimers	Stabilization, self-assembly		Passive + ligand targeting	Controlled release, reduced toxicity	[263]
Surface-functionalized NPs	Surfactants for functional coating		Enhanced tumor uptake	Improved drug loading and tumor targeting	[266]
Liposomal chemotherapeutics	Phospholipid-based surfactants		Targeted HER2 delivery	Enhanced cytotoxicity, reduced side effects	[267]
Multistage DDS	pH-sensitive surfactants		TME-responsive release	Spatiotemporal drug release	[268]
pH-sensitive NPs	pH-responsive surfactants		TME-triggered release	Effective hepatocellular uptake	[269]
Engineered NPs	Amphiphilic surfactants		Ligand/receptor targeting	Precision delivery, biodistribution	[273]
FA-conjugated nanocarriers	FA-modified surfactant		FA-receptor targeting	Enhanced breast tumor specificity	[276]
FA-HA-polymeric micelles	HA and FA surfactants		Dual receptor targeting (CD44, FA)	Paclitaxel delivery, MDR reversal	[278]
Hydrophobized HA micelles	Amphiphilic HA-based surfactants		Dual targeting (CD44, FA)	Enhanced accumulation in resistant cells	[279]
Surfactant–metal complexes (Cu, Co)	Intercalative, DNA-binding		Enhanced hydrophobicity-driven uptake	DNA binding; antimicrobial, anticancer activity	[290,291,292,293,294,295,296,297,298,299,300,301]
siRNA nanoarchitectures	Lipid-based surfactants		MDR gene silencing	Reversal of doxorubicin resistance	[303]
siRNA nanocarriers	Surfactant–lipid hybrids		Gene therapy for MDR	Efficient siRNA delivery	[306]
HER2-targeted NPs	PEGylated surfactants		HER2-targeting antibodies	Enhanced diagnosis and therapy	[312]
pH-sensitive polymeric NPs	Surfactant-assisted micelles		Doxorubicin release in acidic TME	Improved efficacy in MDR tumors	[316,334]
Various surfactants (nanoparticle stabilizers)	Nanoparticles for drug delivery	Improve stability, targeting, and control release	Discusses surfactants in nanoparticle-based drug delivery targeting cancer microenvironment	Targeted cancer therapy	[325]
Brij surfactants	Doxorubicin-loaded nanoparticles	Overcome P-gp mediated efflux, enhance drug retention	Structure–activity relationship of Brij surfactants overcoming drug resistance	Multidrug resistant cancer treatment	[335,336]
Amphiphilic diblock copolymers	Caco-2 cell models	Enhance permeability, inhibit P-gp	Study on polymer-surfactant impact on P-gp substrate permeability	Improve oral bioavailability	[337]
Various pharmaceutical surfactants	Intestinal transport models	Modulate efflux transporters (e.g., P-gp, BCRP)	Effects of surfactants on efflux-mediated drug transport	Oral drug delivery optimization	[338]
Surfactin (biosurfactant)	Nanocarriers for surfactin delivery	Enhance anticancer activity	Anticancer activity of surfactin and nano-assisted delivery	Cancer treatment	[339]
Various surfactants (Tween, Cremophor EL, etc.)	Multidrug-resistant tumor cells	Reverse MDR by inhibiting efflux	Surfactants reversing MDR in cancer cells	Overcome MDR in cancer	[340]
Micellar surfactant systems	Functionalized micelles (targeted)	Improve drug loading, targeting	Development of functionalized micelles for drug delivery	Cancer targeted drug delivery	[341]
Polysorbates, PEG-based surfactants	Nanocarriers	Enhance penetration, inhibit efflux transporters	Advances in surfactant-assisted nanoparticles overcoming MDR	Cancer nanotherapy	[342]

**Table 6:** Surfactant-based nanocarriers for drug delivery and MDR reversal in cancer.

Surfactant	Coated Materials	Role of Surfactant	Description of the Study	Applications	Ref.
TPGS (d-alpha-tocopheryl polyethylene glycol 1000 succinate)	Liposomes, nanoparticles	Inhibits P-glycoprotein (P-gp) by blocking ATPase activity; enhances drug accumulation	TPGS improves intracellular accumulation of drugs like doxorubicin and paclitaxel by inhibiting P-gp activity, leading to reversal of MDR.	Overcoming drug resistance, enhancing chemotherapeutic efficacy	[337,343]
Tween 80	Liposomes, polymeric nanoparticles	Alters membrane fluidity; inhibits P-gp-mediated efflux	Tween 80 enhances drug retention in resistant cancer cells by inhibiting drug efflux, leading to increased cytotoxicity.	Enhancing drug absorption and retention in MDR cancer cells	[344]
Brij 35	Polymeric micelles, nanoparticles	Modifies efflux pump activity, alters membrane fluidity	Brij 35 increases cytotoxicity of chemotherapeutic agents by enhancing cellular drug accumulation and disrupting efflux mechanisms.	Improving effectiveness of chemotherapy in resistant tumors	[338]
TPGS + Chemotherapeutic agents (e.g., paclitaxel)	Mixed micelles, targeted carriers	Solubilizes drugs, inhibits efflux pumps, improves bioavailability	Clinical trials show TPGS–paclitaxel combinations bypass P-gp resistance, improving drug solubility and intracellular retention.	Clinical applications in MDR cancer therapy	[336]
Surfactant–drug complexes (TPGS, Tween 80, Brij 35)	Micelles, liposomes, nanoparticles	Enhances membrane permeability, forms mixed micelles for better uptake	Surfactants form micellar complexes with hydrophobic drugs, increasing drug stability, cellular uptake, and reducing efflux.	Targeted drug delivery, improved drug solubility in resistant cancers	[345,346]
Targeted surfactant-carrier systems (modified surfactants with ligands)	Ligand-conjugated nanoparticles (e.g., folate, EGFR)	Targets tumor-specific receptors, minimizes off-target effects	Surfactant-based carriers are modified with targeting ligands to deliver drugs specifically to tumor cells, improving selectivity and reducing systemic toxicity.	Personalized cancer therapy overcoming MDR	[341,347]

## Data Availability

The datasets generated and analyzed during this study are available from the corresponding author upon reasonable request.
